# Adsorptive separation using self-assembly on graphite: from nanoscale to bulk processes†

**DOI:** 10.1039/d2sc01354a

**Published:** 2022-07-14

**Authors:** Brent Daelemans, Samuel Eyley, Carlos Marquez, Vincent Lemmens, Dirk E. De Vos, Wim Thielemans, Wim Dehaen, Steven De Feyter

**Affiliations:** Division of Molecular Imaging and Photonics, Department of Chemistry, KU Leuven Celestijnenlaan 200F 3001 Leuven Belgium steven.defeyter@kuleuven.be; Division of Molecular Design and Synthesis, Department of Chemistry, KU Leuven Celestijnenlaan 200F 3001 Leuven Belgium wim.dehaen@kuleuven.be; Sustainable Materials Lab, Department of Chemical Engineering, KU Leuven Campus Kulak Kortrijk, E. Sabbelaan 53 8500 Kortrijk Belgium; Centre for Membrane Separations, Adsorption, Catalysis and Spectroscopy for Sustainable Solutions (cMACS), KU Leuven Celestijnenlaan 200F 3001 Leuven Belgium

## Abstract

Adsorptive separation is a promising lower-energy alternative for traditional industrial separation processes. While carbon-based materials have a long history in adsorptive removal of organic contaminants from solution or gas mixtures, separation using an adsorption/desorption protocol is rarely considered. The main drawbacks are the limited control in bulk adsorption experiments, as often all organic molecules are adsorbed, and lack of desorption methods to retrieve the adsorbed molecules. Using high-resolution on-surface characterization with scanning tunneling microscopy (STM), an increased understanding of the on-surface adsorption behavior under different conditions was obtained. The insight obtained from the nanoscale experiments was used to develop a highly selective separation method using adsorption and desorption on graphite, which was tested for the separation of quinonoid zwitterions. These experiments on adsorptive separation using self-assembly on graphite show its potential and demonstrate the advantage of combining surface characterization techniques with bulk experiments to exploit different possible applications of carbon-based materials.

## Abstract 2

Insights from high-resolution on-surface characterization techniques are used to improve the control over adsorption and desorption on graphite in bulk adsorptive separation processes.

## Graphical abstract

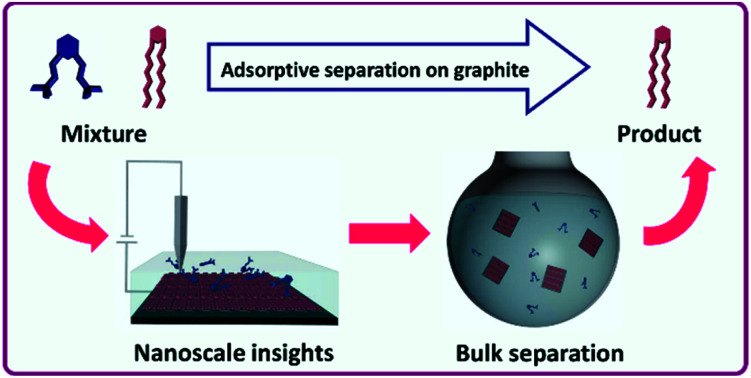

## Introduction

Carbon-based materials have received increasing attention in the last decades as a sustainable and affordable alternative for metals in various applications, including catalysis and electronics.^1–7^ One of the major applications of carbon-based materials is the selective adsorption of molecules in processes, such as water treatment, solvent recovery among others.^8–11^ Although carbon-based adsorbents have been used for centuries, research on the adsorption properties of carbon materials still finds a lot of attention, and many studies focus on the application of the more recently isolated carbon-nanomaterials, including graphene derivatives.^12^ Graphene is a one-atom thick layer of sp^2^-hybridized carbon atoms which was first isolated by Geim and Novoselov in 2004 and still receives substantial attention due to its exceptional properties for a large range of applications.^13^ These properties include high hydrophobicity, large delocalized π–π electron system, and large surface area (up to 2600 m^2^ g^−1^) which make graphene a very potent adsorbent.^14,15^ A main hurdle in the use of graphene in separation technology is its large-scale production. Consequently, more readily accessible graphene derivatives, such as graphene oxide (GO), reduced graphene oxide (rGO), graphene nanoplatelets (GNPs) or graphite, have been used as alternatives.^1,4^ In the recent literature, the adsorptive elimination of different hazardous substances including antibiotics, gases, metals, phenolic compounds and dyes from water using graphene derivatives was widely reported.^12,14,16–26^

**Fig. 1:**
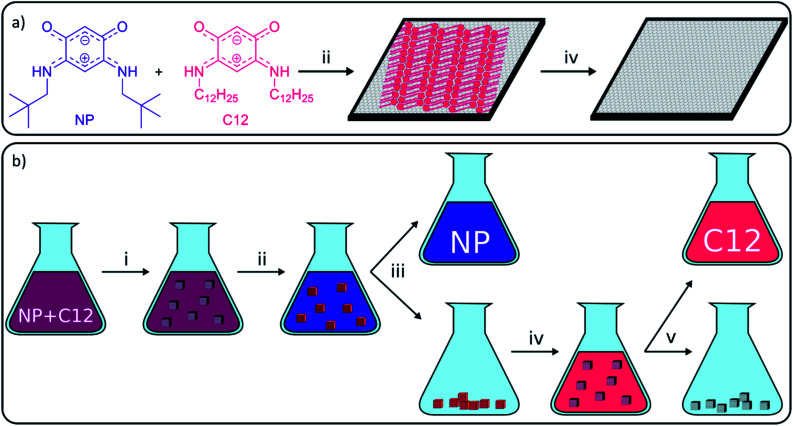
(a) Selective assembly and disassembly of a SAMN of quinonoid zwitterion (QZ) C12 on a graphite surface from a 1/1 mixture of QZ NP (blue) and C12 (red) on the nanoscale, (b) general concept of adsorptive separation in bulk with graphite. Different operations: (i) graphite (grey cubes) is added to a 1/1 mixture of NP and C12, (ii) selective adsorption of compound C12 on graphite, (iii) centrifugation to separate C12 adsorbed on graphite and the solution containing mainly NP, (iv) washing of the graphite to desorb C12, and (v) centrifugation to separate the solution containing C12 and graphite.

While most studies on bulk adsorption with carbon-based materials focus on solution characterization to determine what has been removed from solution, our work will use advanced surface science microscopy and spectroscopy techniques to study the on-surface behavior. These techniques are able to give additional information on adsorption sites and adsorption mechanisms which allows gaining better control over adsorption on carbon-based materials. To establish this, our group uses high-resolution scanning tunneling microscopy (STM), which was developed in 1981 by Binnig and Rohrer,^27^ to characterize the graphite surface and visualize the behavior of adsorbed molecules with submolecular resolution. Common observations on surfaces are the formation of self-assembled monolayers (SAMs), such as alkyl thiols on gold,^28^ or self-assembled molecular networks (SAMNs), such as trimesic acid on graphite.^29^ Both types are typically stabilized by intermolecular interactions, including van der Waals (VDW) interactions, π–π interactions, metal–ligand interactions and hydrogen (H-) bonding. In this work, only the formation of SAMNs on graphite will be discussed. The strength of molecule–substrate and intermolecular interactions differs from molecule to molecule, and therefore the likelihood to adsorb and form SAMNs in a competitive context differs amongst molecules. Molecules successful in forming SAMNs typically contain long alkyl chains and/or large aromatic parts and/or sites for directional non-covalent interactions such as H-bonding. Research groups have been able to use this difference in adsorption energy to separate molecules from a mixture and increase the selectivity of reactions, including Schiff base reaction, olefin metathesis or the synthesis of benzothiazoles.^2,30–35^ While the developed methods are very interesting from a fundamental point of view, all of them describe the separation on the nanometer scale using HOPG and characterization using STM. The importance of these concepts would increase significantly if they could also be applied efficiently in bulk. However, methods to upscale nanoscale experiments on HOPG to bulk experiments on graphitic powders are still lacking. In 2015, our group presented a work where observations on HOPG were linked to the effect of porous graphitic carbon in bulk reactions, which allowed us to explain the observed selectivity.^33^ While an increased rate and selectivity were obtained in the presence of porous graphitic carbon, the yield itself could not be increased as desorption of the molecules was not achieved. Since desorption methods for self-assembled structures on carbon adsorbents are still lacking, our aim was to apply surface characterization techniques to discover conditions to selectively assemble and disassemble SAMNs and to efficiently desorb the molecules from the graphite surface (Fig. 1).

**Scheme 1:**
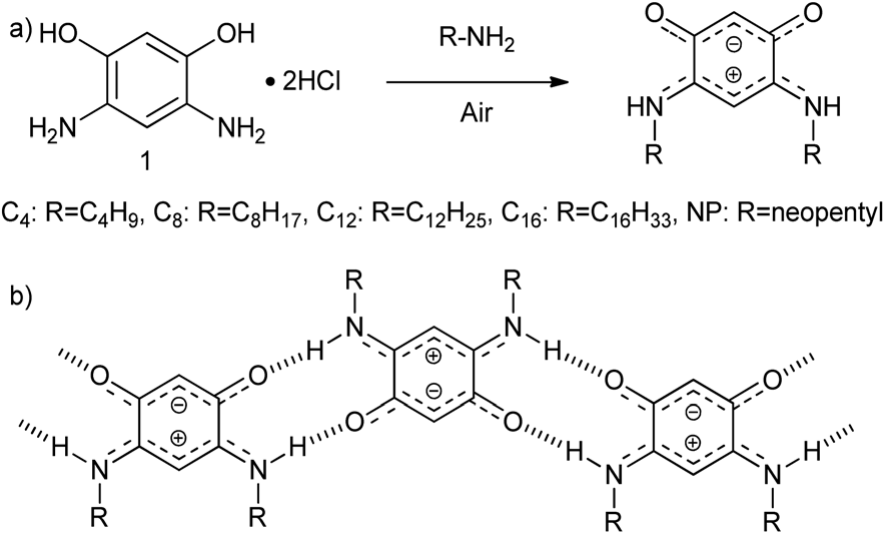
(a) Synthesis of QZ with different chains from 4,6-diaminoresorcinol dihydrochloride (1), (b) primary motif of the self-assembly of QZ with charge-assisted intermolecular hydrogen bonding.

To accomplish this goal, a molecular system with a strong tendency to form SAMNs on graphite was necessary, and we selected quinonoid zwitterions (QZs),^36,37^ more specifically 2,5-diamino-1,4-benzoquinonemonoimine derivatives (Fig. 1a), for reasons that will be discussed further on. 2,5-Diamino-1,4-benzoquinonemonoimine derivatives were first synthesized by Braunstein *et al.* in 2002.^38^ A simpler synthesis method was discovered a couple of years later by the same group, which allowed the synthesis of QZs under ambient conditions using 4,6-diaminoresorcinol and an amine.^39^ These QZs possess many interesting characteristics like a 6π + 6π electron system^40^ or large intrinsic dipoles^41^ and have been used in different applications, including metal-chelating agents^42^ or electronics.^43^ There are two main structural characteristics of the QZ that cause the formation of a self-assembled structure: (i) charge-assisted hydrogen bonding between the hydrogen on the nitrogen and the oxygen of a different QZ, and (ii) VDW interactions between alkyl chains. To optimize these interactions the molecules typically self-assemble in a zig-zag type conformation (Scheme 1b).

This work presents a comparison between the selectivity in STM experiments on the nanometer scale and bulk adsorption experiments for different graphitic powders. An attempt to explain the observed differences in selectivity was done using a thorough characterization of the graphitic powders. Furthermore, a separation method using selective adsorption and desorption on graphitic surfaces was established both on the nanometer scale and in bulk which could reach up to 45% separation yields with a selectivity of 99% from a 1/1 mixture.

## Results and discussion

### Synthesis

To achieve bulk separation of the QZs using selective adsorption on graphite, QZs with different chains were synthesized *via* an oxidative transamination of 4,6-diaminoresorcinol (1) (Scheme 1a). In QZ C4, C8, C12 and C16 unbranched alkyl chains were incorporated, which should stabilize the self-assembled structure due to increased VDW interactions, while in QZ NP bulky neopentyl chains were incorporated, which should destabilize the self-assembled structure due to steric hindrance. For a clear comparison of different alkyl chain lengths, QZ C4–C16 were synthesized by following previously reported procedures.^36,39,43,44^ For the synthesis of the previously reported QZ NP,^38^ a similar procedure was followed with neopentylamine which gave the targeted compound in a 86% yield.

### Preferential adsorption on the nanometer scale

**Fig. 2:**
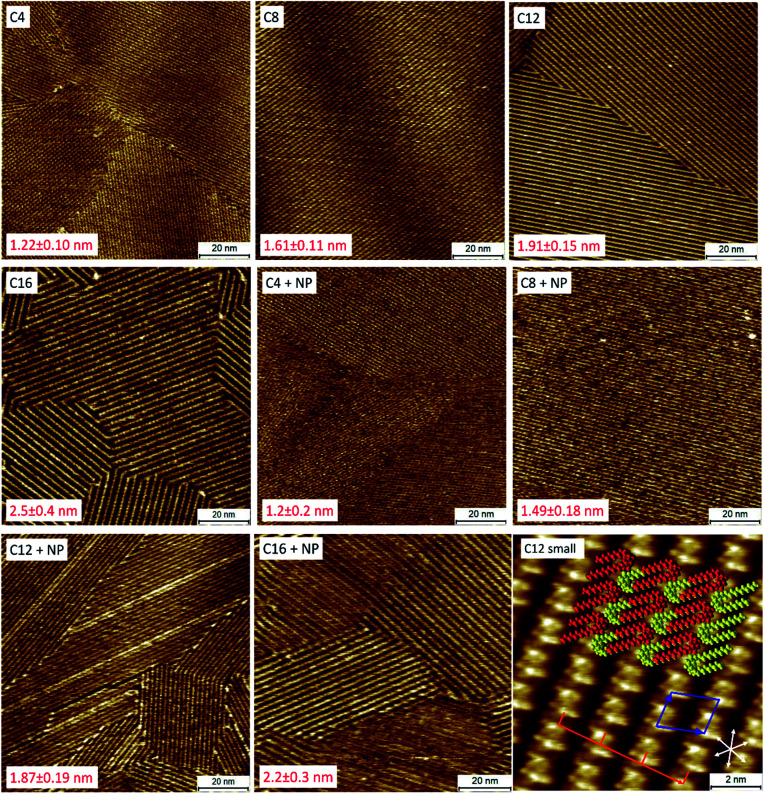
Self-assembly of the QZs at the 1-PO/graphite interface. STM images were obtained from 0.5 mM solutions of pure QZ C4–C16 (C4, C8, C12, and C16) and of 0.25 mM solutions of a mixture of QZ C4–C16 and QZ NP (C4 + NP, C8 + NP, C12 + NP, and C16 + NP). The periodicity and error for each image are indicated in red in the figure and were calculated as the mean and standard deviation over 3 different images with for each image at least 100 measured repeating units. A small-scale image of the SAMN of QZ C12 is shown in C12 small. On this figure, the proposed model with double deck self-assembly (red/yellow), unit cell (blue), periodicity (orange) and graphite axes (white) are indicated. Imaging conditions: 100 × 100 nm^2^ or 10 × 10 nm^2^, 0.020–0.080 nA, −0.800–0.006 V.

The next step was to gain insight in the adsorption behavior of the different QZs on graphite. High-resolution STM experiments were conducted to determine their behavior with molecular precision. In these experiments, two different types of samples were investigated with STM: (i) pure QZs to determine if the different molecules form SAMNs on the surface, and (ii) mixtures of two QZs to determine if one of the molecules would preferentially or selectively adsorb on the surface. For the pure QZ, a 0.5 mM solution was made in 1-phenyloctane (1-PO), a common liquid for STM experiments at the liquid–solid interface. This solvent was chosen because of its low vapor pressure, its electrochemical inertness, and its similarity to toluene, the solvent used for bulk experiments. Next, the solution was drop-casted on a freshly cleaved HOPG platelet and the self-assembled structure was visualized with STM at the liquid–solid interface. For the pure samples, QZs C4–C16 formed SAMNs that could easily be visualized on HOPG (Fig. 2), while a SAMN of the bulky QZ NP was not observed. A representative small-scale STM image of QZ C12 is also shown in Fig. 2 together with the proposed model, which was previously reported in 1,2,4-trichlorobenzene (TCB).^36^ The proposed model indicates the presence of a double-deck self-assembly where the SAMN is stabilized with charge-assisted hydrogen bonding between the head groups and VDW interactions between the alkyl chains. These results indicate the tendency of QZ C4–C16 to form SAMNs on a graphite surface.

Another parameter that can be calculated from the small-scale image in Fig. 2 is the amount of molecules that adsorb per unit area. This parameter is interesting to compare the nature of the adsorption on the nanometer scale and the bulk scale. If the amount of molecules per unit area is similar, then it is possible to conclude that the nature of the adsorption is the same on the nanometer scale and in bulk. If there would be a significantly higher amount of adsorption per unit area on the bulk scale, then formation of a more dense SAMN or the formation of multilayers might occur. Using the area of the unit cell that was observed for the SAMN of QZ C12 (2.63 ± 0.12 nm^2^), the amount of molecules that adsorb per square nanometer can be calculated. Since a unit cell contains two molecules, the number of molecules per unit of area is 0.76 nm^−2^. This value can then be converted to 1.3 μmol m^−2^ using the Avogadro constant. This value will be compared below to the values found for adsorption on graphitic powders to determine the nature of the adsorption on graphitic powders.

After imaging the SAMNs of the pure compounds, the self-assembly of 1/1 mixtures of the bulky QZ NP with the different QZs C4–C16 (final concentration of each QZ is 0.25 mM) was investigated with STM (Fig. 2). If these images are compared to the images of the pure mixtures, the periodicities of the SAMNs are closely in agreement which indicates that a self-assembled structure is formed, identical to the one formed by QZs C4–C16. The periodicities were measured perpendicular to the rows of the self-assembled head groups of the QZs, which is indicated by the orange line in the small-scale image. The periodicities were measured for three images on different positions with at least 100 repeating units for each sample for which the mean and standard deviation were calculated. The selective adsorption of QZs C4–C16 can be explained by the steric hindrance that is created by the bulky neopentyl groups which inhibits the formation of SAMNs. In the case of the unbranched QZs, the alkyl chains do not cause any steric hindrance but rather stabilize the self-assembled structure *via* VDW interactions between the different chains, and between the chains and the graphite surface. As the periodicities were calculated over images at different locations on the HOPG surface, we can conclude that in the mixtures only the unbranched molecules adsorb on the basal plane of graphite.

### Upscaling to bulk experiments

While separation of two QZs can be achieved in a 100% selective way *via* adsorption on a HOPG surface, upscaling to bulk conditions is necessary to increase the practical value of this separation method. There are numerous differences between experiments on nanometer and bulk scale which means that several challenges need to be faced to make both experiments comparable. The three main challenges are: (i) the characterization of adsorbed molecules as STM cannot be used for bulk powders, (ii) the development of a desorption protocol to recover the adsorbed molecules from the surface, and (iii) the choice of the graphitic powder for bulk experiments, taking into account the presence of different defects and functional groups (mainly oxygen containing).^45–48^ These different groups affect the adsorption properties of the material, for example H-bond donor adsorption sites will strongly adsorb H-bond acceptor molecules through the formation of H-bonds.^19,49,50^ Before adsorptive separation experiments can be performed, solutions for these three challenges should be found.

### Graphitic powders

**Scheme 2:**
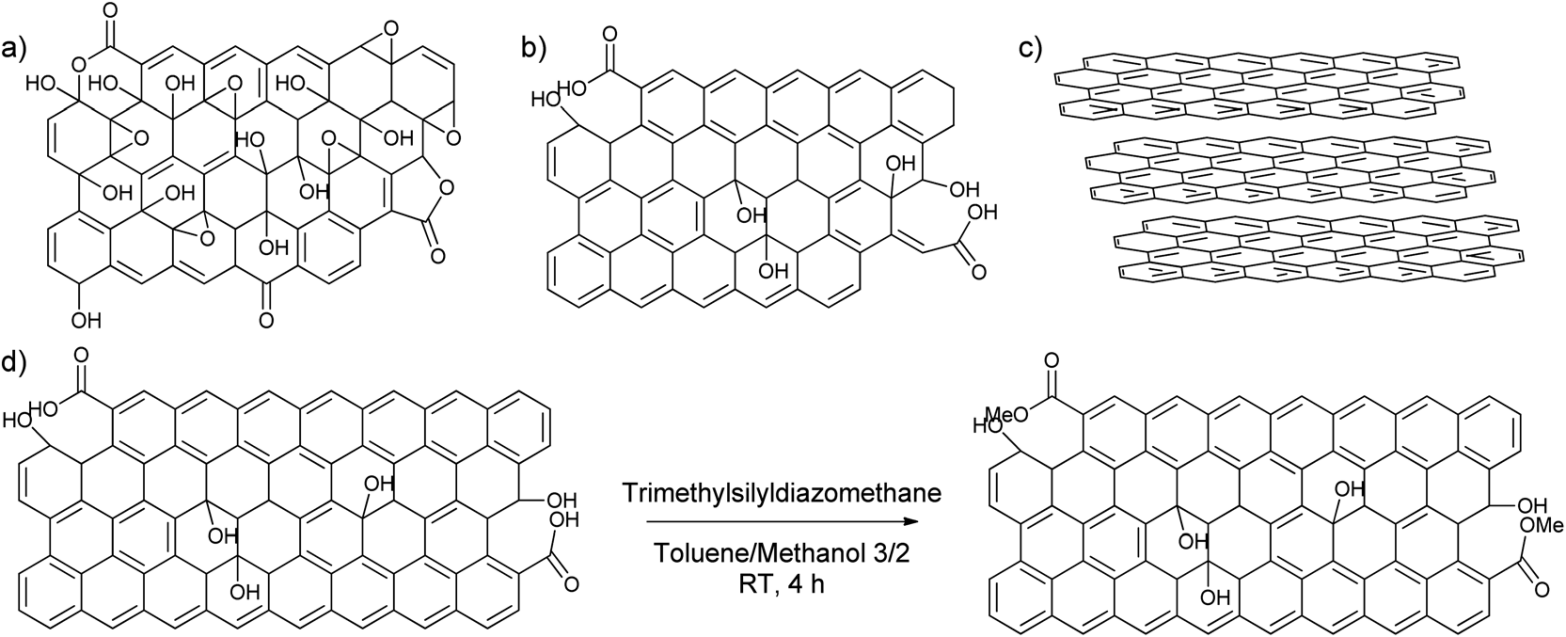
(a) GO, (b) rGO, (c) graphite, (d) deactivation from GNP to deactivated GNP with trimethylsilyldiazomethane. Reaction conditions: 1 g GNP, 5 mL 2 M trimethylsilyldiazomethane in diethylether, 200 mL toluene/methanol 3/2, RT, 4 hours.

To find out the most suitable powder for adsorptive separation, different graphitic powders were considered containing variable amounts of defects and oxygen functionalities. The selected graphitic powders are GO, rGO, GNP and graphite. Furthermore, since the GNP still contain a large amount of oxygen-containing functional groups, an attempt was done to reduce the amount of active oxygen adsorption sites by reacting the powder with trimethylsilyldiazomethane (Scheme 2). In this way, the carboxylic acid groups that could form strong hydrogen bonds are methylated to reduce their hydrogen bonding capacities. The success of the deactivation was determined using a Boehm titration with NaOH where the total amount of acidic groups was reduced from 0.238 ± 0.003 mmol g^−1^ for GNP to 0.112 ± 0.012 mmol g^−1^ for deactivated GNP.^47,51^ These powders were then characterized using various techniques to be able to link the nature of the carbon-materials with their adsorption properties.

From the many available techniques to determine the nature of carbon powders, the first chosen characterization technique is N_2_-physisorption. This technique allows one to determine the surface area of the chosen powders. In Table 1, it is shown that GO and graphite have a very low surface area compared to the rest of the samples, 18.5 m^2^ g^−1^ for GO and 6 m^2^ g^−1^ for graphite in comparison to 480 m^2^ g^−1^ for rGO, 760 m^2^ g^−1^ for GNP, and 625 m^2^ g^−1^ for deactivated GNP. Furthermore, GO and graphite were found to exhibit Type II isotherms, which are given by the physisorption of N_2_ on microporous or nonporous adsorbents, while rGO, GNP and deactivated GNP were found to exhibit isotherms between Type II and Type IV, which are given by mesoporous adsorbents (Fig. S9, ESI†).^52–56^

**Table 1:** Properties of the different carbon-materials determined with N_2_-physisorption, Raman spectroscopy, and XPS

	GO	rGO	GNP	Deact. GNP	Graphite
Surface area (m^2^ g^−1^)	18.5 ± 0.3	480 ± 3	760 ± 6	625 ± 5	5.9 ± 0.2
*I*_D_/*I*_G_	3.35 ± 0.14	3.78 ± 0.10	3.49 ± 0.12	4.54 ± 0.10	0.21 ± 0.03
*I*_D′_/*I*_D_	0.14 ± 0.02	0.11 ± 0.01	0.12 ± 0.01	0.10 ± 0.02	0
Carbon content (at%)	70.4 ± 0.3	88.6 ± 0.7	91.8 ± 1.2	90.5 ± 1.1	96.2 ± 1.6
Oxygen content (at%)	28.6 ± 0.2	11.3 ± 0.7	7.6 ± 1.2	8.9 ± 1.1	3.4 ± 1.5
Sulfur content (at%)	0.72 ± 0.07	—	—	—	—

The second chosen characterization technique is Raman spectroscopy which was used to compare the amount of defects between the graphitic powders. The different powders can be compared by using their *I*_D_/*I*_G_ ratios (Table 1). For accurate comparison of the ratios, the peaks were deconvoluted in the D (∼1330 cm^−1^), D* (∼1500 cm^−1^), G (∼1585 cm^−1^), and D′ (∼1620 cm^−1^) bands (Fig. S10, ESI†). The D band is associated with the order/disorder of the system while the G band is an indicator of the stacking structure. The ratio of the area of both bands (*I*_D_/*I*_G_ ratio) gives more information on the degree of disorder and the degree of exfoliation where a high *I*_D_/*I*_G_ ratio indicates a high degree of exfoliation/disorder. An interesting observation is the increase in the *I*_D_/*I*_G_ ratio of the reduced graphitic materials (rGO or GNP) in comparison to GO. One might expect a decrease in this ratio as the amount of oxygen functional groups decreases. However, the reduction from GO to rGO or GNP also creates sp^3^-hybridized carbon atoms that reduce the size of the sp^2^-domains and increase the *I*_D_/*I*_G_ ratio.^57^ The other bands in the Raman spectrum can also give an additional information about the material. The D* band is related to amorphous phases where its intensity decreases when the crystallinity increases and the D′ band is ascribed to highly defective layers. It has been reported previously that the areas of the D′ peak and D peak (*I*_D′_/*I*_D_ ratio) can be used to interpret the types of defects present in the graphitic material,^58,59^ where an *I*_D′_/*I*_D_ value of 0.143 corresponds to vacancy-like defects and an *I*_D′_/*I*_D_ value of 0.077 to sp^3^ defects.^60^ The *I*_D′_/*I*_D_ ratios for the different materials are shown in Table 1. While GO seems to be predominated by vacancy-like defects, rGO, GNP, and Deact. GNP have contributions from both vacancy-like defects and sp^3^-defects, for graphite, no D′ peak was observed. Common defects that induce disorder in the basal plane include carboxylic acid groups, epoxy, and hydroxyl groups.

The presence of the oxygen functional groups was confirmed using X-ray photoelectron spectroscopy (XPS), which is the final chosen characterization technique. XPS is able to determine the content of different atoms in the material. This technique indicates the presence of large amounts of oxygen present in the powders, from 3.4% for graphite up to 28.6% for GO (Table 1 and Fig. S11, ESI†). The large amount of oxygen means that there is a significant difference with the basal plane of HOPG where the amount of oxygen functional groups is negligible. Furthermore, sulfur is also observed on GO in XPS which can be attributed to the presence of sulfate groups that were created under the strongly oxidizing preparation conditions, such as the Hummers' method.^61^ While many other characterization techniques for carbon powders exist, these three characterization techniques were deemed essential to explain the adsorption behavior of the QZs on different graphitic powders.

### Characterization of the adsorbed molecules

**Fig. 3:**
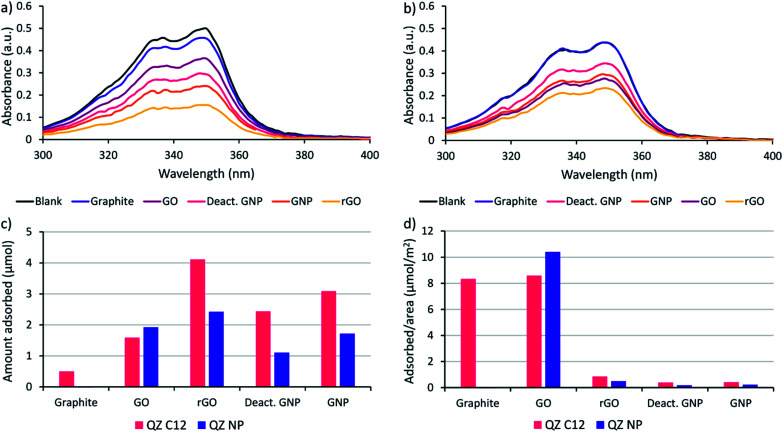
Decrease in absorbance *via* adsorption of (a) QZ C12 and (b) QZ NP on different graphitic powders, (c) amount of adsorption of a pure solution of QZ C12 or NP on different graphitic powders ordered from left to right following increasing surface area, and (d) amount of adsorption per unit area of the graphitic powders. Adsorption conditions: 24 h, 60 °C, 3 mM QZ, 2 mL toluene, 10 mg powder.

Before performing the adsorptive separation experiments with graphitic powders, a characterization and a desorption method for the adsorbed molecules in bulk need to be established. While STM can determine the surface composition on HOPG locally, this technique does not work for bulk graphitic powders. Hence, a new method to determine the amount and nature of the adsorbed molecules was required. Since the QZs absorb light in the visible region, UV/VIS spectroscopy is a possible method to determine the total amount of QZ adsorbed. To determine the feasibility of this method, the adsorption of pure QZ C12 and QZ NP was tested for the different graphitic powders. Solutions with a 3 mM concentration of the pure QZ were stirred in toluene in presence of 10 mg of the graphitic powders for 24 hours at 60 °C. A significant decrease in absorbance could be observed for every powder with QZ C12, while with QZ NP no decrease was observed for graphite (Fig. 3a and b).

From the difference in absorbance between the blank and the solutions that were stirred in presence of a powder, the adsorbed amount can be calculated (Fig. 3c). If the adsorbed amount on different graphitic powders is compared for QZ C12, the powders with a high surface area (rGO, GNP and deactivated GNP) show a higher amount of adsorption than GO or graphite. However, the difference is smaller than expected from the differences in surface area. This observation can be seen more clearly when the amount of adsorption is corrected for the surface area (Fig. 3d). The lower amount of adsorption/area for graphitic powders with high surface areas could be caused by an incomplete coverage of the surface. Additionally, N_2_-physisorption could underestimate the effect of the oxygen functionalities which could explain the significantly higher adsorption of rGO than GNP or deactivated GNP. When the amount of adsorption per unit area for graphite in bulk (8.3 μmol m^−2^) is compared to the amount that was observed on HOPG (1.3 μmol m^−2^), the amount of adsorption per unit area in bulk is significantly higher. This observation suggests the formation of multilayers on graphite under the used adsorption conditions. The same adsorption experiment was repeated with QZ NP and some clear differences in the adsorption of QZ C12 and NP are observed: (i) the least oxygenated graphitic powder has practically no adsorption of QZ NP, (ii) the strongly oxygenated GO has a higher adsorption of QZ NP than of QZ C12 while a clear decrease in adsorption is observed for all other powders. These observations indicate that the nature of the carbon material can have a significant effect on the adsorption of different QZs, which will be discussed in more detail below.

### Desorption of the adsorbed molecules

**Fig. 4:**
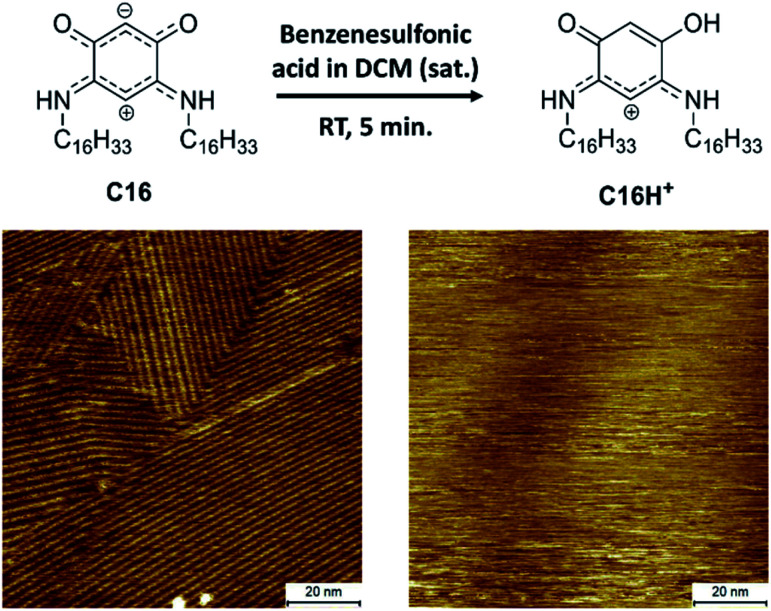
Desorption of QZ C16 from the graphite basal plane using protonation with a strong acid. Imaging conditions: 100 × 100 nm^2^, 0.5 mM in 1-PO, 0.060 nA, −0.800 V.

To be able to desorb the adsorbed molecules and obtain adsorptive separation, a washing method was developed which allows disassembly of the SAMNs of the QZs and washes the molecules from the surface. A possible way to disassemble the SAMNs would be to inhibit the intermolecular interactions. While it would be difficult to inhibit VDW interactions, the QZs can easily be modified to lose their hydrogen bonding capacities by protonating them with a strong acid.^40^ Using this information, a washing method with dichloromethane (DCM) and benzenesulfonic acid was considered. This washing method was first tested on HOPG and followed by STM and XPS. The SAMN of QZ C16 in 1-PO was visualized with STM (Fig. 4), followed by a washing step where the HOPG was immersed in a saturated solution of benzenesulfonic acid in DCM and dried. QZ C16 was chosen for this experiment, as it is expected to form the most stable SAMN of QZ C4–C16 as the VDW interactions are maximized. After the washing step, a droplet of 1-PO was added and the surface was visualized again with STM where no self-assembled structure could be observed anymore.

In addition to the STM experiments, XPS analysis of both the unwashed and washed HOPG samples confirmed the removal of QZ C16 from the surface. The removal of QZ C16 is observed by the significantly lower nitrogen 1s peak in the XPS spectrum for the washed HOPG (0.08 at%) compared to the unwashed sample (1.1 at%) (Table S2, ESI†). This washing step verifies that it is possible to gain control over the adsorption and desorption of molecules on graphite using simple chemical manipulations. In combination with the 100% selective adsorption of QZ C4–C16, this washing step should allow us to use self-assembly and disassembly on the surface as a separation method of two quinonoid zwitterions.

### Selective adsorption in bulk experiments

**Fig. 5:**
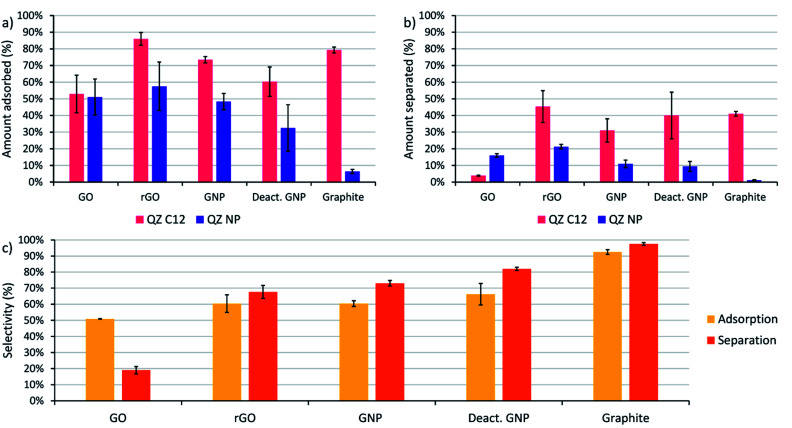
(a) Amount of adsorption of QZ C12 and QZ NP on different graphitic powders ordered from left to right following decreasing oxygen content, (b) amount of molecules separated after adsorption and desorption on graphitic powders, (c) influence of the graphitic powder on the adsorption selectivity (yellow), which is defined as the amount of adsorbed QZ C12 divided by the total amount of adsorbed molecules (C12 + NP), and the separation selectivity (orange), which is defined as the amount of QZ C12 in the separated solution divided by the total amount of QZ in the separated solution (C12 + NP). The value and error for each experiment were calculated as the mean and standard deviation over at least 3 different experiments. Adsorption conditions general (graphite): 24 h, 60 °C, 1.5 (0.15) mM of both QZ C12 and NP, 2 (20) mL toluene, 10 (500) mg powder. Desorption conditions: 3× washed with a 5 mL sat. solution of benzenesulfonic acid in DCM at RT.

The next step is to extend the preferential adsorption observed at the nanoscale towards a possible separation method using the previously discussed graphitic powders. To accomplish this, 2 mL of a 1/1 mixture of compounds C12 and NP both in a 1.5 mM concentration in dry toluene was added to 10 mg of the different graphitic powders. Toluene was chosen as a solvent because of its similarity to the STM solvent 1-PO. For the experiments with graphite, only a small amount of adsorption was observed with 10 mg powder (Fig. 3c) due to its small surface area and the reaction conditions were changed to 20 mL of a 0.15 mM solution with 500 mg of powder to allow a significant amount of adsorption. While GO also has a small surface area, the amount of adsorption was significant and no adapted conditions were necessary. The mixtures were stirred for 24 hours at 60 °C to allow the adsorption equilibrium to be reached. Afterwards, the graphitic powders were removed by centrifugation and washed with toluene. The QZ that stayed in solution were characterized using UV/VIS, which allows accurate characterization of the total QZ concentration, and ^1^H-NMR, which allows to determine the QZ C12/QZ NP ratio that is left in solution, and therefore also the amount and QZ C12/QZ NP ratio adsorbed on the graphitic powders (Fig. 5a).

The experiments using adsorption of a pure solution of a QZ on different graphitic materials indicated that QZ NP has a higher amount of adsorption on more oxygenated graphitic powders. Next, our aim is to determine how the nature of the substrate influences the selectivity of the adsorption from a 1/1 mixture. The effect of the graphitic powder on the amount of adsorption of both QZ is shown in Fig. 5a, where a clear decrease of adsorption of QZ NP is observed for decreasing oxygen content. However, except for graphite the errors on the amount of adsorption are always significant. These errors could be due to differences in functional groups present on different fractions of the powder. These errors can be corrected by changing to the adsorption selectivity, which is defined as the amount of adsorbed QZ C12 divided by the total amount of adsorbed molecules (C12 + NP), and which corrects the errors by comparing the adsorption of both molecules. The adsorption selectivity is shown in yellow in Fig. 5c, in which substrates with lower amounts of active oxygen groups, such as graphite or deactivated GNP, are observed to reach a higher adsorption selectivity while the strongly oxygenated GO is not selective at all. These observations indicate that the presence of oxygen functional groups is a cause of non-selective adsorption of QZ on graphitic powders. According to literature, a possible mechanism for this non-selective adsorption can be attributed to H-bonding on the polar sites on the graphitic powders.^16,62–64^ These observations show that the presence of oxygen groups has a significant effect on the adsorption selectivity and that the 100% selectivity that is reached on the pure basal plane on HOPG, as visualized in STM, cannot be reached with the oxygen containing graphitic powders.

Graphite is observed to have the most similar adsorption behavior to HOPG, with a large amount of adsorption on basal plane sites and a low amount of adsorption on oxygen functionalities. The amount of adsorbed QZ per unit area can be compared to the value on HOPG to determine the nature of adsorption on the graphitic powders. The amount of adsorption per unit area can be calculated by dividing the amount of adsorbed QZ (2.55 μmol) by the added surface area (3 m^2^) which gives a value of 0.85 μmol m^−2^. The amount of adsorption per unit area in these experiments is lower than the value that was determined on HOPG (1.3 μmol m^−2^), which suggests that under these conditions a monolayer structure is present and that the graphite surface of the powder is not completely saturated with the formed SAMN.

### Selective separation in bulk experiments

After determination of the adsorption behavior of the QZs on different substrates, washing of the adsorbed molecules from the surface was attempted to determine if adsorptive separation on graphite was possible. The procedure that was tested on HOPG was upscaled to bulk conditions and the graphitic powders were washed three times with a saturated solution of benzenesulfonic acid in DCM. The solution that was washed from the surface will be called separated solution below. After washing three times with 5 mL of saturated benzenesulfonic acid in DCM, the separated solution was characterized using UV/VIS and ^1^H-NMR to determine the amount of QZ present and the QZ C12/QZ NP ratio. In Fig. 5c, the orange columns show the separation selectivity, which is defined as the amount of QZ C12 in the separated solution divided by the total amount of QZ in the separated solution (C12 + NP). If the separation selectivity of the different powders is compared, the same relation between selectivity and presence of oxygen functionalities is observed. In the case of graphite, QZ C12 could be separated with a separation selectivity of 98% after one adsorption/desorption step, which makes graphite the most effective graphitic powder for adsorptive separation in these experiments.

A disadvantage of graphite is the low surface area which limits the sustainability of the separation process as much solvent is required to wash the graphitic powder. In this experiment, 15 mL of solvent was used to wash off 1.26 μmol of QZ which means that the separated solution had a concentration of 0.0842 mM. To increase the sustainability of the adsorption process, most researchers focus on increasing the surface area of graphitic powders to increase the amount of molecules adsorbed. However, our experiments show that it does not necessarily improve their properties for adsorptive separation. Because of its high purity, the low-surface area graphite gives the best results in our experiments. From our observations, we can conclude that the ideal graphitic powder for adsorptive separation using assembly and disassembly of SAMNs should have a high surface area together with a low amount of oxygen functionalities.

When the adsorption and separation selectivity are compared, the separation selectivity is generally higher than the adsorption selectivity. This difference can be explained by strong H-bonding to certain functional groups on the surface. The graphitic powders contain different H-bonding sites, including carboxylic acids, phenols, and epoxides, that will have different interaction strengths with the QZs. On GO, even some sulfate groups are present which can be observed by the sulfur peak in XPS and which were created due to the synthesis of GO under strongly oxidizing conditions. A part of the QZs will attach to those strong H-bonding sites and will be too strongly bound to the graphitic powder to be removed by the washing step. This strong bonding can also explain why there is a significant difference between the amount of molecules adsorbed and the amount of molecules separated. As H-bonding is not selective, the molecules that stay on the oxygen functionalities will have approximately a 1/1 distribution. Since the adsorbed molecules are enriched in QZ C12, removal of a 1/1 amount of QZ C12 and NP will remove a lower percentage of QZ C12 than of QZ NP and increase the amount of QZ C12 in the separated solution even more. In this way, the molecules that stay on the oxygen functionalities will increase the separation selectivity in comparison to the adsorption selectivity.

### Recycling experiments

**Fig. 6:**
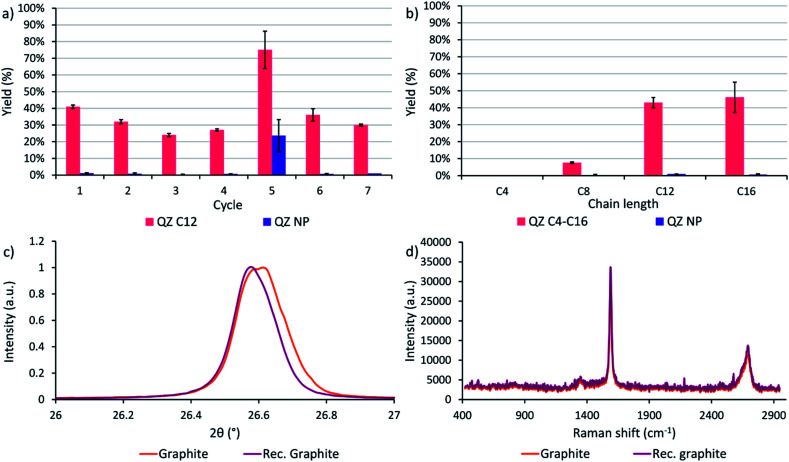
(a) Recycling experiments using the same graphitic powder. The yield is defined as the amount of QZ C12 or NP in the separated solution divided by the initial amount of respectively QZ C12 or NP in the mixture. Cycle 1 uses the commercial graphite powder, cycle 2 uses a 1× recycled powder. The powder is neutralized in between cycles with methanol/triethylamine (95/5). (b) Effect of the chain length on the yield. The value and error for each experiment were calculated as the mean and standard deviation over three different experiments. Reaction conditions: 24 h, 60 °C, 0.15 mM of both QZs, 20 mL toluene, 500 mg powder. (c) (002) peak of the commercial graphite and the recycled graphite in XRD. (d) Raman spectra of the commercial graphite and the recycled graphite.

After determining that graphite is the most suitable graphitic powder for our adsorptive separation experiments, the powder was tested in a recycling experiment. In cycle 1 with the commercial powder, a yield of 41% was achieved where the yield is defined as the amount of QZ C12 or NP in the separated solution divided by the initial amount of respectively QZ C12 or NP in the mixture. Next, the graphitic powder was neutralized after the washing step with methanol/triethylamine (95/5) and tested again under the same conditions. In these experiments, a downwards trend of the yield is observed while the separation selectivity stays high (Fig. 6a). After 4 cycles, the graphitic powder shows a different behavior where a significantly higher yield but lower selectivity is obtained after the separation. A possible explanation for the different behavior in cycle 5 could be intercalation/exfoliation, where the QZs slowly intercalate over time beneath the top layer and eventually cause an exfoliation of this layer. While in general sonication is used for intercalation/exfoliation graphite, stirring over very long times might have a similar effect. Intercalation in graphite can be observed with multiple techniques, such as X-ray diffraction (XRD) and Raman spectroscopy. In XRD, a shift to lower angles of the (002) peak at ∼26.6° corresponds to an increase of the interplanar distance between neighboring layers in graphite and shows the presence of intercalation. In Raman spectroscopy of graphite, a very small D band at ∼1350 cm^−1^ and a 2D band at ∼2700 cm^−1^ consisting of two component peaks are typically observed, while for intercalated graphite the bands change to a larger D band and a broad 2D band consisting of one peak.^65^ However, neither a clear shift to lower angles of the (002) peak nor a difference in the Raman spectrum were observed (Fig. 6c and d), which contradicts the presence of intercalation in the graphitic powder. Further investigation is necessary to find the origin of this outlier. In the sixth and seventh cycle, a high separation selectivity is obtained again. These experiments show that graphite can be recycled efficiently without losing its high separation selectivity.

### Effect of the chain length

A final separation experiment was to determine the effect of the chain length on the yield and separation selectivity. On HOPG, compounds C4–C16 are observed to adsorb with 100% selectivity with regard to QZ NP. However, extending the preferential adsorption to graphite powder might influence the formation of self-assembled structures on the surface and thus the adsorption and separation selectivity due to slightly different conditions, such as a different surface area, concentration, and temperature. When the four different chain lengths are compared, a clear increase in the separation yield is observed when going to longer chain lengths C12 and C16 (Fig. 6b). A yield of 41% and 45% and a separation selectivity of 98% and 99% were obtained for QZ C12 and C16 respectively, while for C8 a yield of 8% and a selectivity of 96% was obtained and for C4 no separation was observed. This increase can be explained by the increase in VDW interactions in the SAMN when going to longer chain lengths. The increase in intermolecular interactions stabilizes the SAMN and allows a higher amount of adsorption and separation for QZ with longer chain lengths under bulk conditions.

## Conclusions

So far, most research on adsorptive separation focused on the adsorption on zeolites, metal organic frameworks or covalent organic frameworks. Carbon materials, such as graphite, were rarely considered because of the poor control over adsorption and more importantly, lack of reliable desorption methods. This work succeeded in increasing the control and determining desorption methods using high-resolution on-surface characterization with STM. Using STM, we determined differences in adsorption selectivity and showed which conditions are necessary to disassemble the SAMNs and desorb the molecules. Afterwards, the experiments were successfully upscaled to bulk conditions using different graphitic powders. While the presence of oxygen groups on graphitic powders does not allow to reach 100% selectivity in bulk, a separation selectivity of 99% and yield of 45% could be achieved with adsorptive separation on graphite starting from a 1/1 mixture. Furthermore, the effect of the chain length was determined where a clear increase in separation yield was found for longer chain lengths. This trend originates from the increasing VDW interactions and thus stronger SAMNs for longer chain lengths. Finally, the recyclability of graphite was determined where the graphite powder showed a small decrease in separation yield over time.

This work on separation using assembly and disassembly of SAMNs shows the potential of adsorptive separation on graphite and demonstrates the advantage of combining on-surface characterization techniques with bulk experiments to exploit different possible applications of carbon-based materials. To fully exploit the potential of graphite in bulk experiments, there are still some challenges that need to be tackled. A first challenge would be the production of graphitic powders with a higher purity and larger surface areas. As shown in this work, the oxygen functional groups can have a strong effect on the adsorption properties and significantly lower the selectivity. A larger surface area would limit the solvent necessary for the washing of the graphitic powder and increase the sustainability of the separation process. A second challenge is to develop more methods to allow desorption of molecules from graphite. While our method using protonation and cleavage of the hydrogen bonds is feasible for certain molecules, those molecules are only a small part of the molecules that can self-assemble on graphite. In this regard, different methods making use of other functionalities or molecular properties should be developed. The final challenge is to use this selective adsorption to influence reactions. While the effect of the basal plane on reactions has been widely studied in nanoscale experiments,^2^ the upscaling to bulk conditions was rarely attempted. This work shows that the upscaling from nanoscale to bulk scale experiments can be done under static conditions. However, the upscaling might be a lot more challenging under dynamic conditions. A similar combination of on-surface characterization and bulk experiments might be key in tackling these challenges and further developing carbocatalysis.

## Data availability

Data for this paper, including XPS, Boehm titration, Raman, N_2_-physisorption, XRD, STM, NMR and UV/VIS, are available at the KU Leuven research data repository at https://doi.org/10.48804/KVUSSH.

## Author contributions

Conceptualization: B. D., W. D., S. D.; methodology: B. D.; investigation: B. D., S. E., C. M., V. L.; supervision: D. D., W. T., W. D., S. D.; writing-original draft: B. D.; writing-review and editing: B. D., S. E., C. M., V. L., W. D., S. D.

## Conflicts of interest

There are no conflicts to declare.

## Supplementary Material

SC-013-D2SC01354A-s001
