# Supplementary material for: Adsorptive separation using self-assembly on graphite: from nanoscale to bulk processes
Source: Chem Sci. 2022 Jul 14;13(31):9035–46. doi: 10.1039/d2sc01354a (PMC9365087; doi:10.1039/d2sc01354a)
Supplement: SC-013-D2SC01354A-s001 [file SC-013-D2SC01354A-s001.pdf]

Electronic Supplementary Material (ESI) for Chemical Science.
This journal is © The Royal Society of Chemistry 2022

Adsorptive separation using self-assembly on graphite: from nanoscale to bulk processes

Brent Daelemans[1,2], Samuel Eyley[3], Carlos Marquez[4], Vincent Lemmens[4], Dirk E. De Vos[4], Wim Thielemans[3], Wim Dehaen[2] and Steven De Feyter[1]

[1]*Division of Molecular Imaging and Photonics, Department of Chemistry, KU Leuven, Celestijnenlaan 200F, 3001 Leuven, Belgium*

[2]*Division of Molecular Design and Synthesis, Department of Chemistry, KU Leuven, Celestijnenlaan 200F, 3001 Leuven, Belgium*

[3]*Sustainable Materials Lab, Department of Chemical Engineering, KU Leuven, campus Kulak Kortrijk, E. Sabbelaan 53, 8500 Kortrijk, Belgium*

[4]*Centre for Membrane Separations, Adsorption, Catalysis and Spectroscopy for Sustainable Solutions (cMACS), KU Leuven, Celestijnenlaan 200F, 3001 Leuven, Belgium*

Contents:

1. Ex-situ synthesis of quinonoid zwitterions

    General experimental procedure

    Synthesis of 4,6-di(neopentylamino)-m-quinone (QZ **NP**)

    Reference ^{1}H-NMR spectra of QZ **NP** (Figure S1)

2. Self-assembly of pure QZ **C4-C16** and mixtures with QZ **NP** at the 1-phenyloctane/HOPG interface and disassembly under acidic conditions

    General experimental procedure

    STM visualizations (Figure S2-S8)

3. Deactivation and characterization of different graphitic powders

    General experimental procedure

    Synthesis of deactivated GNP

    Boehm titration

    N_2-physisorption (Figure S9)

    Raman spectroscopy (Table S1, Figure S10)

    X-ray photoelectron spectroscopy (Table S2, Figure S11)

    X-ray diffraction analysis

4. Extinction coefficients from UV/VIS experiments

    General experimental procedure

    UV/VIS spectra and extinction coefficients of different QZ (Figure S12-S21)

5. Bulk adsorption and separation experiments

    General experimental procedure

    Adsorption of pure compounds on graphitic powders

    Adsorption of mixtures on GO, rGO, GNP and deactivated GNP

    Adsorption of mixtures on graphite

    Determination of the adsorption selectivity (Figure S22-S23)

    Determination of the separation selectivity (Figure S24-S25)

    Recycling experiments

    Summary of the obtained results (Table S3-S4)

1. Ex-situ synthesis of quinonoid zwitterions

General experimental procedure

The quinonoid zwitterions **C4**, **C8**, **C12**, and **C16** were obtained according to the procedures reported in the literature.[1-4] 4,6-diaminoresorcinol dihydrochloride (**1**) (98%, Acros Organics NV), *n*-butylamine (99.5%, Acros Organics NV), *n*-octylamine (99%, Acros Organics NV), dodecylamine (98%, Acros Organics NV), hexadecylamine (90%, VWR International BVBA) and neopentylamine (97%, Acros Organics NV) were bought from commercial suppliers. NMR spectra were acquired in $CDCl_3$ on a Bruker AMX 400 MHz and chemical shifts (δ) are reported in parts per million (ppm) referenced to tetramethylsilane (1H).

Synthesis of 4,6-di(neopentylamino)-*m*-quinone (QZ NP):

To a solution of 4,6-diaminoresorcinol dihydrochloride (**1**) (250 mg, 1.173 mmol) in 5 mL water in a 10 mL round bottom flask (RBF) was added neopentylamine (924 mg, 10.6 mmol). The mixture was stirred for 2 hours at room temperature. The precipitate was obtained using a filtration, washed with cold water and dried overnight under reduced pressure to give QZ **NP** (279 mg, 86%) as a red solid. ^{1}H-NMR (400 MHz, $CDCl_3$, Me_4Si): δ 8.40 (s, 2H, NH), 5.51 (s, 1H, OCCHCO), 5.19 (s, 1H, NCCHCN), 3.16 (d, 4H, NCH_2), 1.07 (s, 18H, $C(CH_3)_3$) in accordance with the previously reported data.[5]

Reference ^{1}H-NMR spectra of QZ NP (400 MHz, $CDCl_3$, Me_4Si):

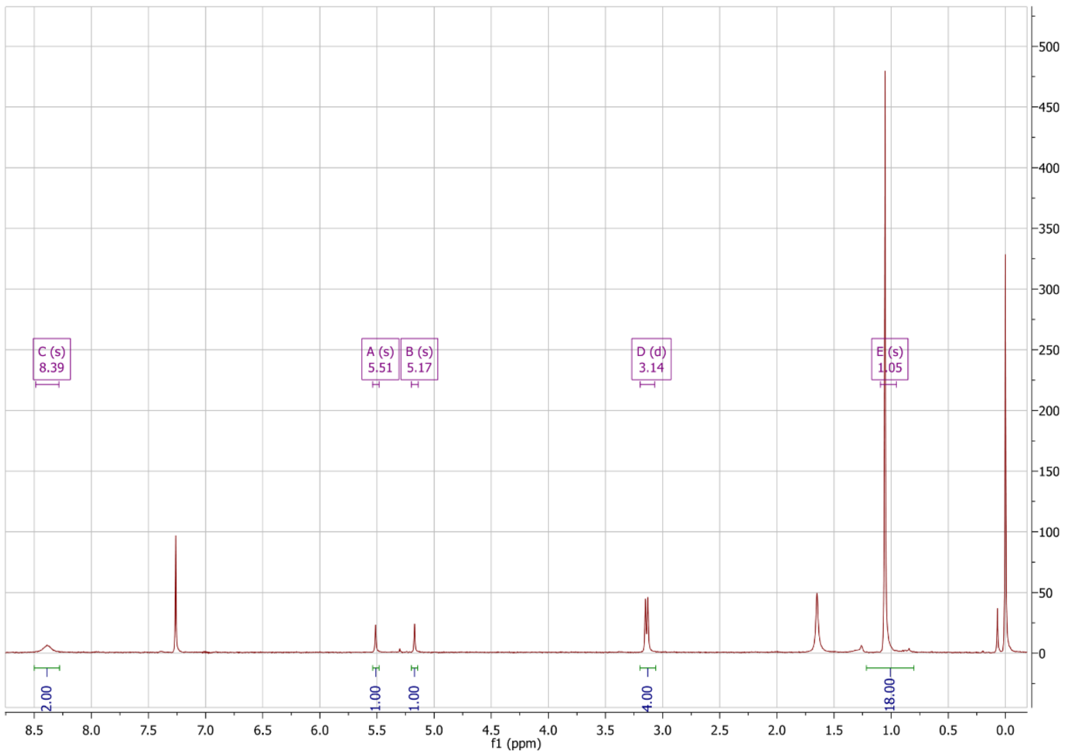

Figure S1. *^{1}H-NMR spectra of QZ **NP** in $CDCl_3$*

2. Self-assembly of pure QZ C4-C16 and mixtures with QZ NP at the 1-phenyloctane/HOPG interface and disassembly under acidic conditions

General experimental procedure

The molecules were dissolved in 1-phenyloctane (1-PO) (Sigma Aldrich) at the given concentrations. All samples were subsequently drop-casted on freshly cleaved HOPG (HOPG, grade ZYB, Advanced Ceramics Inc., Cleveland, OH, U.S.A.). Scanning Tunneling Microscopy (STM, Pico SPM, Agilent) measurements were performed in constant current mode at the liquid-solid interface at room temperature (20-25 °C). Mechanically cut Pt/Ir wire (80/20, 0.25mm diameter) were used as STM tips. Analysis of the unit cell was performed after drift correction by using SPIP software (Image Metrology A/S). Periodicities were averaged over at least 300 measured distances from 3 different images. The error given is the standard deviation calculated over these 300 distances. To visualize the efficiency of the washing protocol, the self-assembled structure of QZ **C16** was first visualized in STM. Next, the HOPG platelet was placed in a saturated solution of benzenesulfonic acid in DCM for 5 minutes at room temperature. The platelet was then dried and visualized with STM after adding one new droplet of 1-phenyloctane.

Self-assembly of QZ C4 and C4+NP

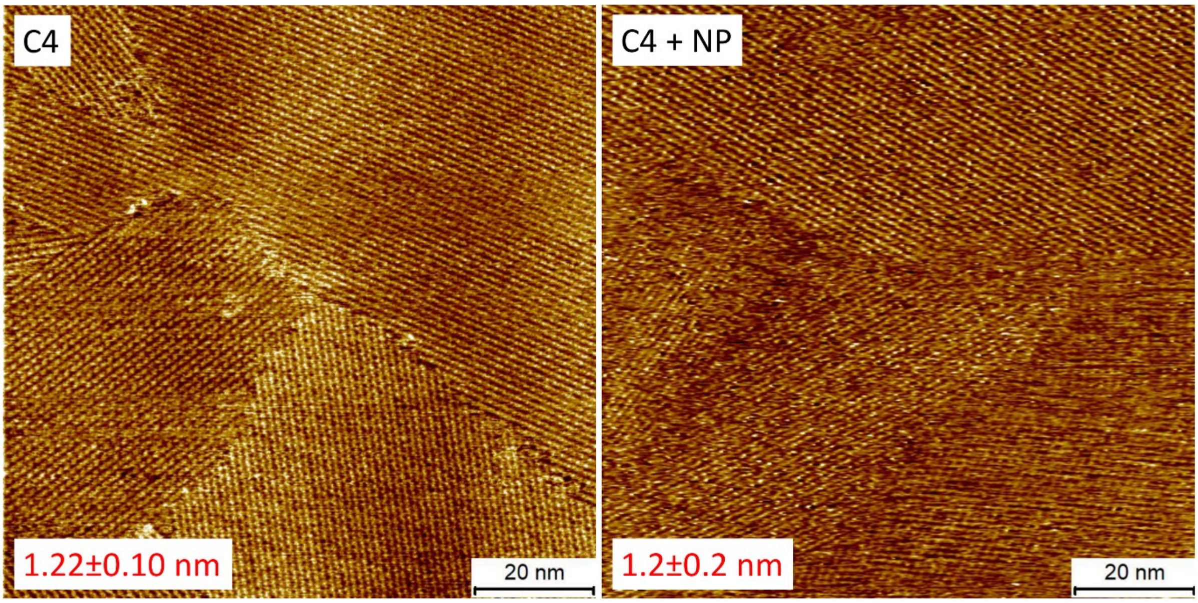

Figure S2. *STM image of QZ **C4**, 100×100 nm², 0.5 mM in 1-PO, 0.020 nA, 0.006 V, periodicity: 1.22±0.10 nm (left) and QZ **C4+NP**, 100×100 nm², 0.25 mM in 1-PO, 0.050 nA, -0.250 V, periodicity: 1.2±0.2 nm (right).*

Self-assembly of QZ C8 and C8+NP

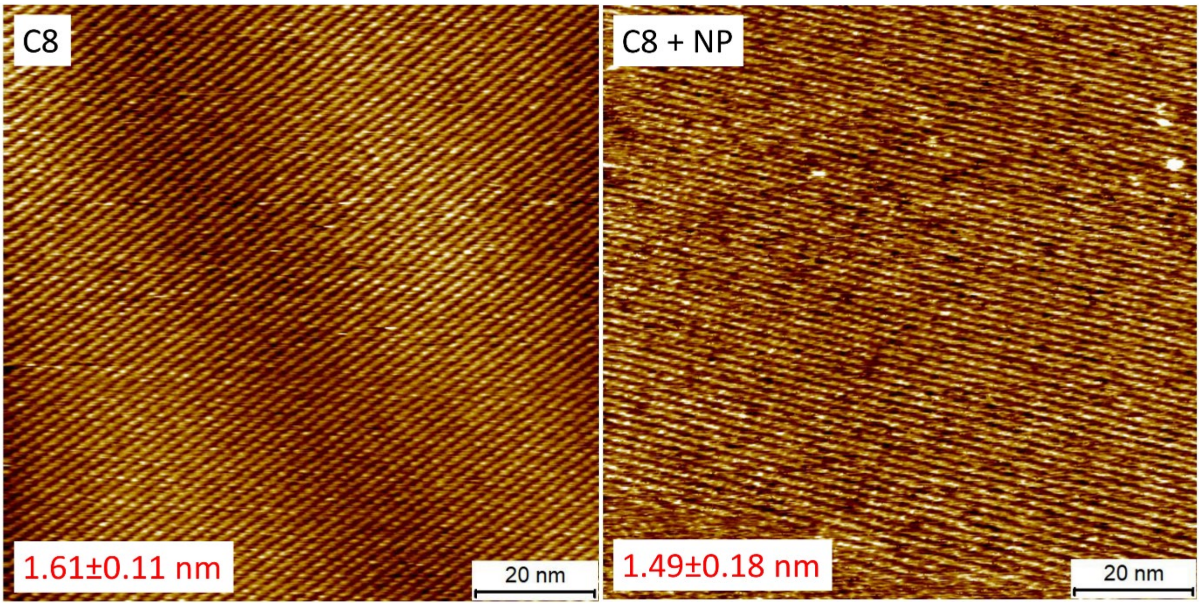

Figure S3. *STM image of QZ **C8**, 100×100 nm², 0.5 mM in 1-PO, 0.050 nA, -0.250 V, periodicity: 1.61±0.11 nm (left) and QZ **C8+NP**, 100×100 nm², 0.25 mM in 1-PO, 0.050 nA, -0.250 V, periodicity: 1.49±0.18 nm (right).*

Self-assembly of QZ C12 and C12+NP

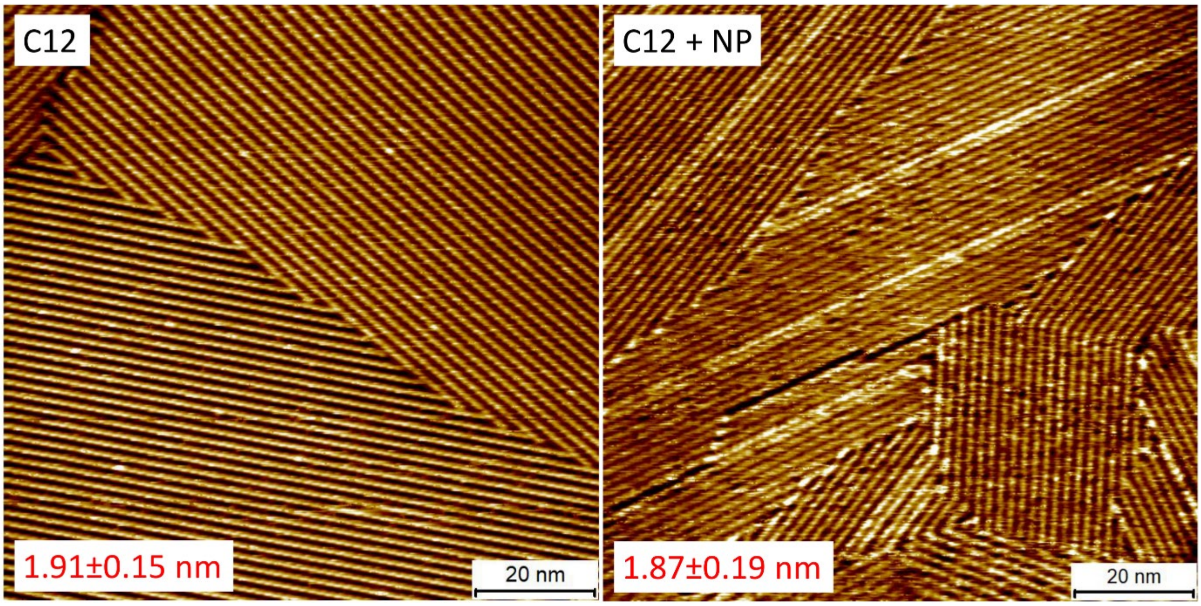

Figure S4. *STM image of QZ **C12**, 100×100 nm², 0.5 mM in 1-PO, 0.080 nA, -0.800 V, periodicity: 1.91±0.15 nm (left) and QZ **C12+NP**, 100×100 nm², 0.25 mM in 1-PO, 0.080 nA, -0.800 V, periodicity: 1.87±0.19 nm (right).*

Self-assembly of QZ C16 and C16+NP

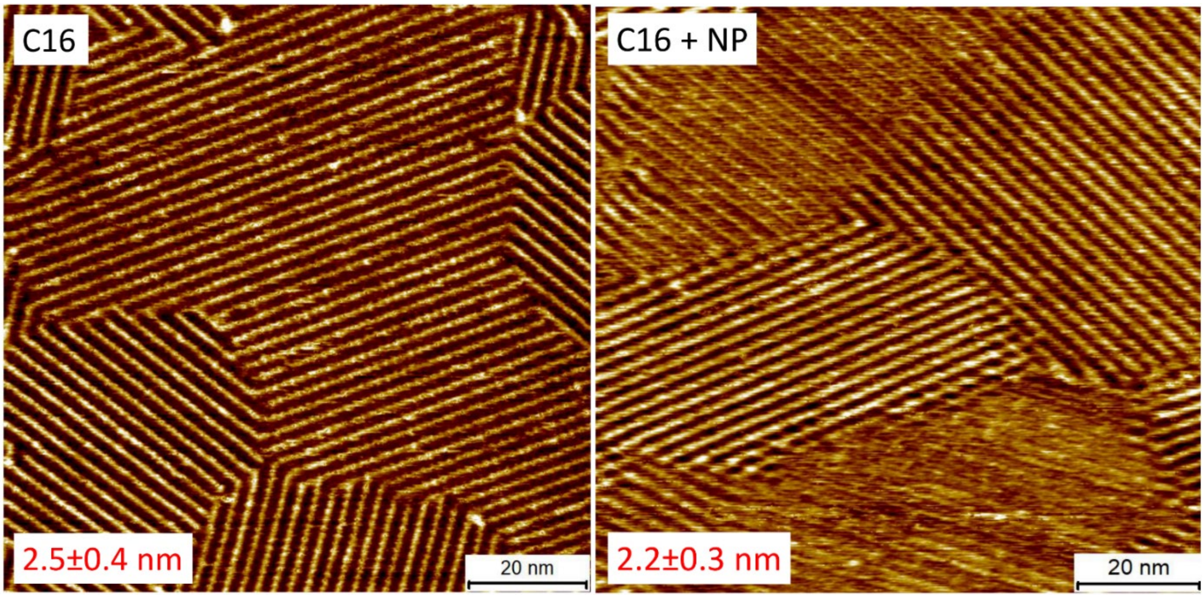

Figure S5. *STM image of QZ* ***C16****, 100×100 nm², 0.5 mM in 1-PO, 0.060 nA, -0.800 V, periodicity: 2.2±0.3 nm (left) and QZ* ***C16+NP****, 100×100 nm², 0.25 mM in 1-PO, 0.050 nA, -0.250 V, periodicity: 2.5±0.4 nm.*

STM visualization of QZ NP

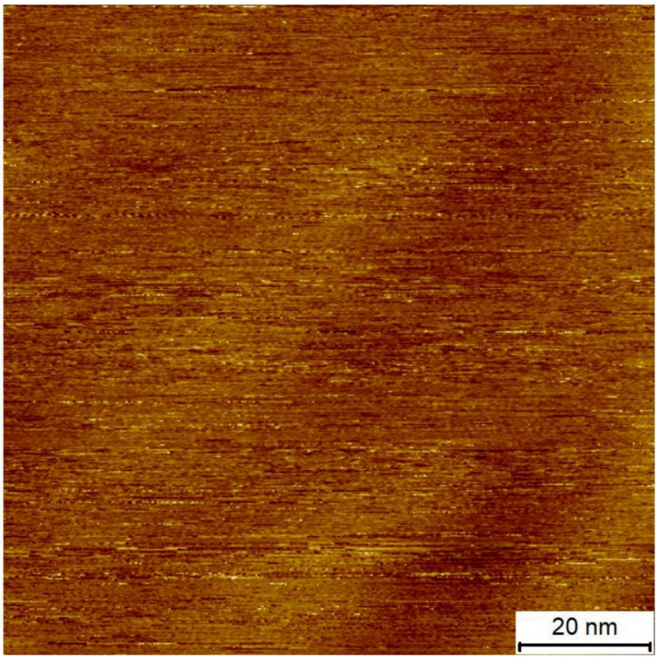

Figure S6. *STM image of a dropcasted solution of QZ* ***NP*** *where no SAMN was observed, 100×100 nm², 0.5 mM in 1-PO, 0.080 nA, -0.800 V.*

High-resolution image BD-QZ-11

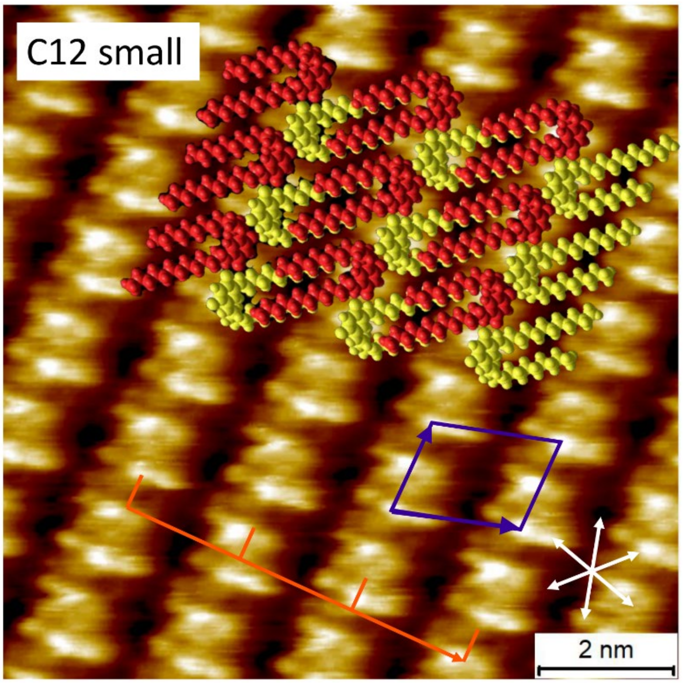

***Figure S7**. High resolution STM image of QZ **C12,** including added model (green and yellow), unit cell (blue) and periodicity (red). Imaging conditions: 10×10 nm^2, 0.5 mM in 1-PO, 0.080 nA, -0.800 V. Unit cell parameters determined over eight images: a=1.43±0.06 nm, b=1.92±0.03 nm, α=74.3±1.6°, area= 2.63±0.12 nm^2*

Self-assembly and disassembly of QZ C16 using washing under strong acidic conditions

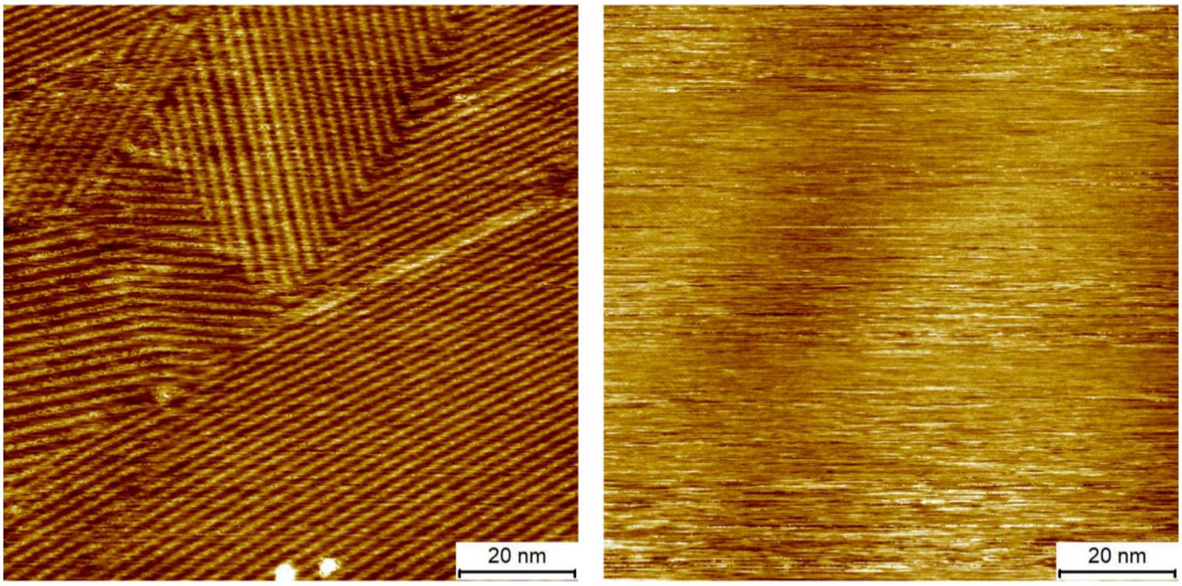

***Figure S8**. STM images of compound **C16**, 100×100 nm^2, 0.5 mM in 1-PO, 0.060 nA, -0.800 V, before washing with DCM+benzene sulfonic acid (left) and after washing (right). During washing the sample was immersed for 5 minutes in a saturated solution of benzenesulfonic acid at RT.*

3. Deactivation and characterization of different graphitic powders

General experimental procedure

GO (49-56 at.% C) and rGO (80-87 at.% C) were purchased from Graphenea. GNP (91 at.% C) was obtained from PlasmaChem and graphite was obtained from Alfa Aesar (APS 2-15 micron, 99.9995% metals based purity).

Synthesis of deactivated GNP

1 gram of GNP was dispersed in 200 mL toluene/methanol 3/2 and sonicated for 30 minutes. 5 mL of a 2M trimethylsilyl diazomethane solution in diethyl ether was added dropwise. The reaction mixture was stirred for 4 hours at room temperature. The reaction was placed in ice, quenched with 5 mL acetic acid and stirred for 30 min at room temperature. The powder was filtered and washed with water+methanol, acetone and dichloromethane. The powder was dried in a vacuum oven for 20 hours at 40 °C.

Boehm titration

0.100 g of GNP and deactivated GNP were dispersed in 40 mL of a 0.05 M NaOH solution. The dispersion was stirred for 24 hours at RT. The graphitic powders were filtered and three aliquots with a volume of 10 mL were taken from the filtrate. Each sample was acidified using 15 mL of a 0.05 M HCl solution for complete neutralization. The acidified solution was purged with N_2 under magnetic stirring for CO_2 expulsion. The aliquots were then titrated using an Excellence T5 automatic titrator (Mettler Toledo, potentiometric titration) with a 0.1 M KOH solution which was standardized with KHP.[6]

N_2-physisorption

N_2 physisorption isotherms (77 K) were collected on a Micromeritics 3Flex Surface Analyzer. Prior to the experiment, the samples were evacuated at 423 K for 16 h. The specific surface area (S_{BET}) was determined using the BET method (0.05–0.3 p/p°).

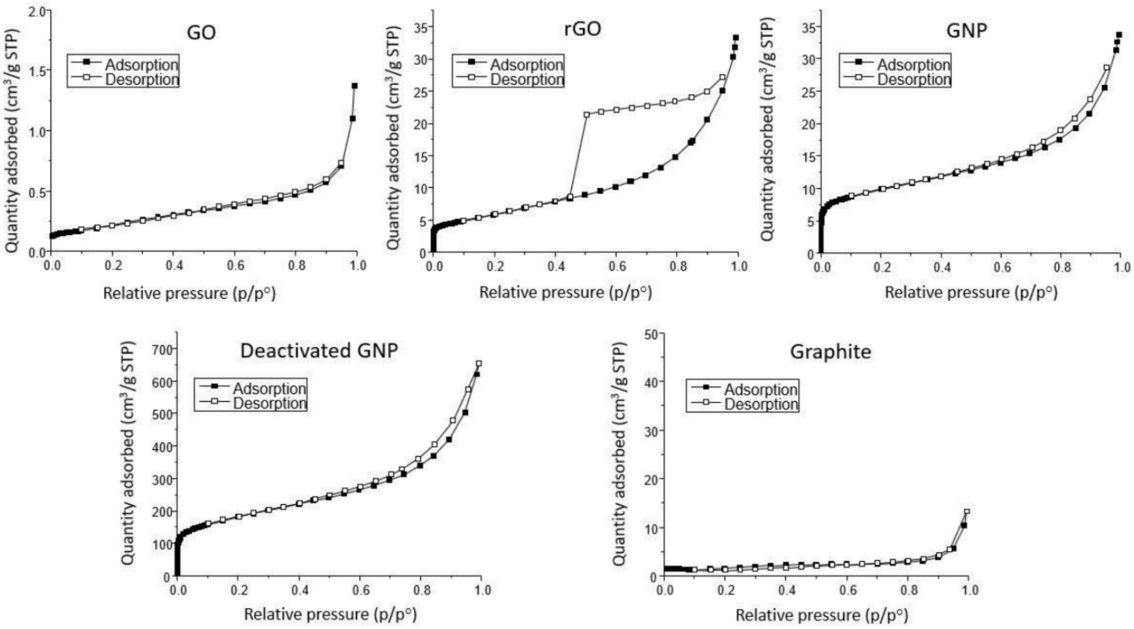

***Figure S9**. N_2-physisorption isotherms of the different graphitic powders.*

Raman spectroscopy

The graphitic powders were dispersed in deionized water (1 mg mL^{-1}) and dropcasted on a silicon wafer. Raman spectra were collected with a confocal Raman microscope (Monovista CRS+, S&I GmbH) using a 632.8 nm He–Ne laser directed on the surface through an objective (OLYMPUS, BX43 100x, N.A. 0.7) with an optical density at the sample surface of about 590 kW cm^{-2}. The Raman scattering was collected using the same objective and guided to a Raman spectrograph (S&I GmbH) equipped with a cooled-charge coupled device (CCD) camera operated at -100 °C (Andor Technology, DU920P-BX2DD). Raman spectra were taken in at least 5 different positions on the sample. All measurements were carried out under ambient conditions at room temperature. Accumulation time for all spectra was 250 s. The Raman spectra were fitted to sums of functions using Origin 9.8 software. Four Lorentz functions were used for deconvolution of the Raman peaks for respectively D (~1330 cm^{-1}), D* (~1500 cm^{-1}), G (~1585 cm^{-1}), and D' (~1620 cm^{-1}) bands. While a D'' around ~1150-1200 cm^{-1} was also expected, this peak was not significant enough to observe during deconvolution. The values that were observed during the Raman measurements are depicted in Table S1. The areas of the peaks were used for calculation of the I_D/I_G and $I_D/I_{D'}$ ratios.

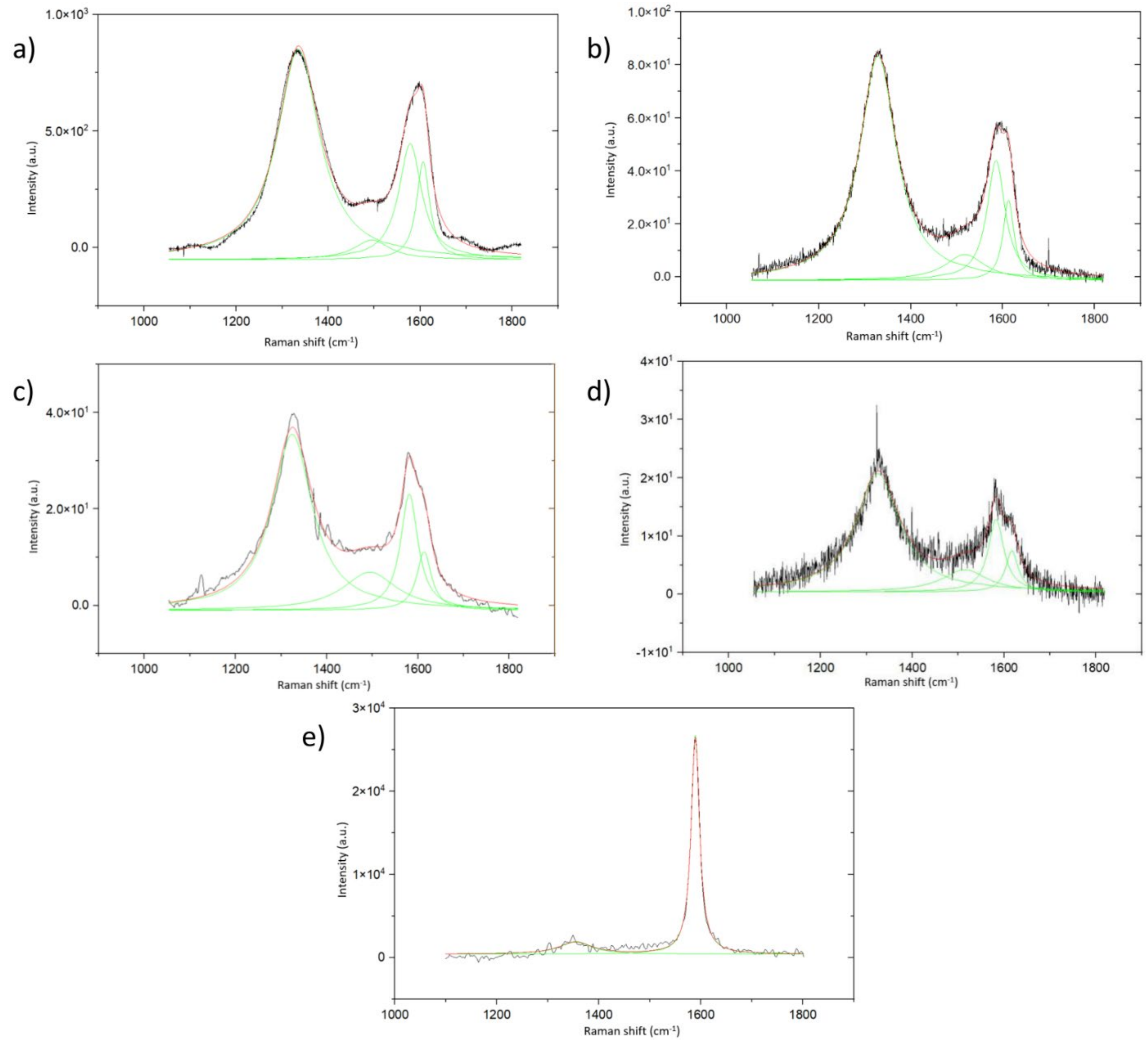

***Figure S10**. Raman spectra of the different graphitic powders a) GO, b) rGO, c) GNP, d) Deactivated GNP, and e) Graphite. Accumulation of different spectra is shown for each sample. Four Lorentz functions were used for deconvolution of the Raman spectra for respectively D (~1330 cm^{-1}), D* (~1500 cm^{-1}), G (~1585 cm^{-1}), and D' (~1620 cm^{-1}) bands.*

***Table S1.** Summary of the discussed values that were obtained in Raman spectroscopy*

powder	***Pos(D)*** ***cm^{-1}***	***I(D)*** ***a.u.***	***Pos(D*)*** ***cm^{-1}***	***I(D*)*** ***a.u.***	***Pos(G)*** ***cm^{-1}***	***I(G)*** ***a.u.***	***Pos(D')*** ***cm^{-1}***	***I(D')*** ***a.u.***
GO	*1335*	*158 10^3*	*1495*	*12 10^3*	*1578*	*47 10^3*	*1606*	*23 10^3*
rGO	*1329*	*13 10^3*	*1518*	*1606*	*1586*	*3399*	*1613*	*1369*
GNP	*1324*	*6152*	*1494*	*1781*	*1581*	*1761*	*1613*	*743*
Deact. GNP	*1325*	*3664*	*1514*	*120*	*1583*	*808*	*1617*	*366*

Graphite	*1354*	*179*<br>*10³*	/	/	*1589*	*822*<br>*10³*	/	/

X-ray photoelectron spectroscopy

X-ray photoelectron spectra were collected using a Kratos Axis Supra photoelectron spectrometer with a monochromated Al Kα X-ray source (hν = 1486.7 eV, 75 W). Samples were mounted in electrical contact with the spectrometer. Spectra were recorded using hybrid (electrostatic/magnetic) optics with a slot aperture (700 µm × 300 µm). The analyzer was operated in fixed analyzer transmission (FAT) mode with survey scans taken at 160 eV pass energy.

Data analysis was performed using CasaXPS version 2.3.25rev1.0Y.[7] Peaks were integrated using a U 2 Tougaard background according to $T(E) = \int_E^\infty F(E' - E)S(E')dE'$, where S(E') is the measured spectrum, $F(x) = \frac{Bx}{(C+x^2)^2}$, C is a user adjustable parameter, and B is automatically adjusted to make the background meet the data at the limits of the interval over which the background is computed.[8] These peak areas were converted to equivalent homogeneous composition[9] using relative sensitivity factors (RSFs) based on Scofield photoelectron cross-sections with angular distribution correction for a source-analyser angle of 60°. Escape-depth correction was performed using the electron attenuation length according to Seah.[10] Data was corrected for the instrument intensity-energy response function (Q(E), equation 2) using an NPL transmission function.[11] This combination of relative sensitivity factors and escape depth correction assumes homogeneous composition within the information depth of the XPS experiment. No corrections were performed for the potential presence of overlayer contamination or matrix effects.

$$Q(E) = \frac{2171.9705 - 757.94045\varepsilon + 299.9256\varepsilon^2 - 464.62852\varepsilon^3 - 98.497537\varepsilon^4}{1 + 0.070275\varepsilon + 0.065215\varepsilon^2 + 0.376419\varepsilon^3 + 0.802171\varepsilon^4} \quad (2)$$

$$\varepsilon = \frac{E - 1000}{1000}$$

Table S2. *Summary of the equivalent homogeneous composition according to XPS for different graphitic samples*

	orbital	RSF	GO	rGO	GNP	Deact. GNP	Graphite	HOPG QZ C16	HOPG washed
**Carbon content (at%)**	C 1s	1.00	70.4 ±0.3	88.6 ±0.7	91.8 ±1.2	90.5 ±1.1	96.2 ±1.6	90.9 ±0.4	97.3 ±1.4
**Oxygen content (at%)**	O 1s	2.93	28.6 ±0.2	11.3 ±0.7	7.6 ±1.2	8.9 ±1.1	3.4 ±1.5	5.4 ±0.5	1.2 ±1.1
**Sulfur content (at%)**	S 2p	1.60	0.72 ±0.07	/	/	/	/	0.48 ±0.05	0.15 ±0.14
**Nitrogen content (at%)**	N 1s	1.80	/	/	0.45 ±0.05	0.4 ±0.3	0.09 ±0.08	1.1 ±0.2	0.08 ±0.05
**Chlorine content (at%)**	Cl 2p	2.18	/	0.08 ±0.03	0.13 ±0.04	0.14 ±0.04	/	0.01 ±0.01	1.0 ±0.3
**Silicon content (at%)**	Si 2P	0.77	0.31 ±0.07	0.06 ±0.06	0.04 ±0.06	/	0.25 ±0.11	2.1 ±0.2	0.2 ±0.3

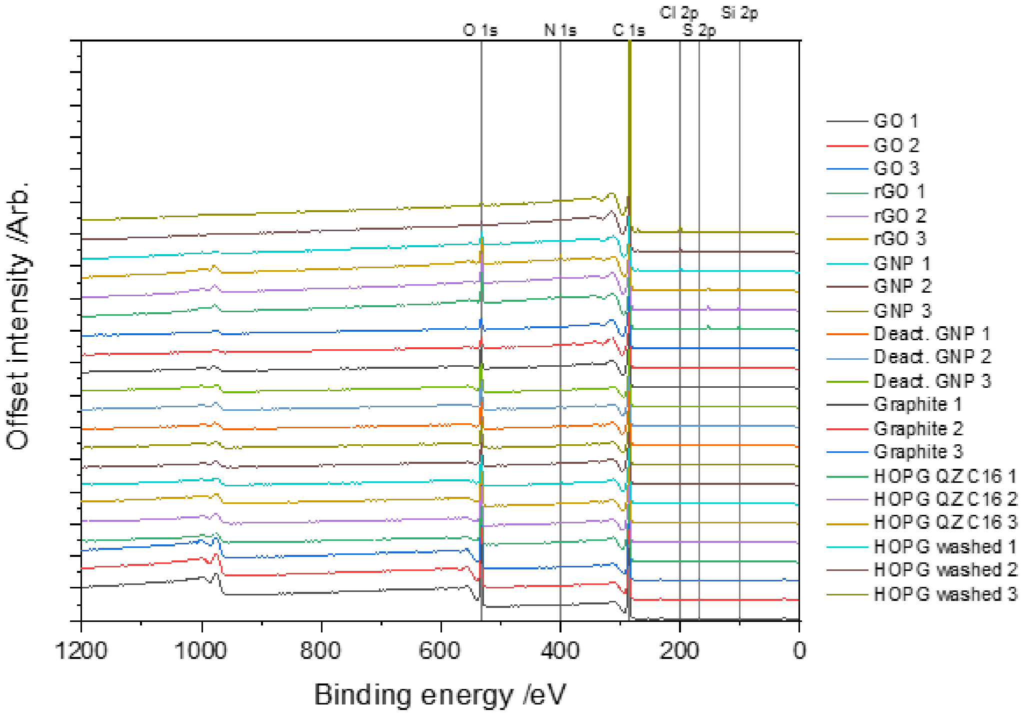

***Figure S11**. XPS survey spectra used for the determination of the elemental composition of the samples.*

X-ray diffraction analysis

Powder X-ray diffraction (PXRD) spectra were recorded on a Malvern PANalytical Empyrean diffractometer. The measurements were carried out in Debye-Scherrer transmission geometry, operating at 45 kV and 40 mA. The X-rays were focused on the sample by means of a curved mirror, which removes all Cu K_{β} radiation and only leaves a beam consisting mainly of Cu $K_{\alpha 1}$ (λ = 1.5406 Å) and to lesser extent of Cu $K_{\alpha 2}$. A solid state PIXcel 3D 1x1 detector records the data within a 1.3°-45° 2θ-range with a step size of 0.013°. The spectra were analyzed via PANalytical Data Viewer software.

4. Extinction coefficients

General experimental procedure

UV/VIS absorption spectra were recorded on a PerkinElmer Lambda950 spectrophotometer using blank correction. Dye solutions were prepared using commercially available dichloromethane and toluene of at least spectro or HPLC grade.

Spectrum and extinction coefficient of QZ C4

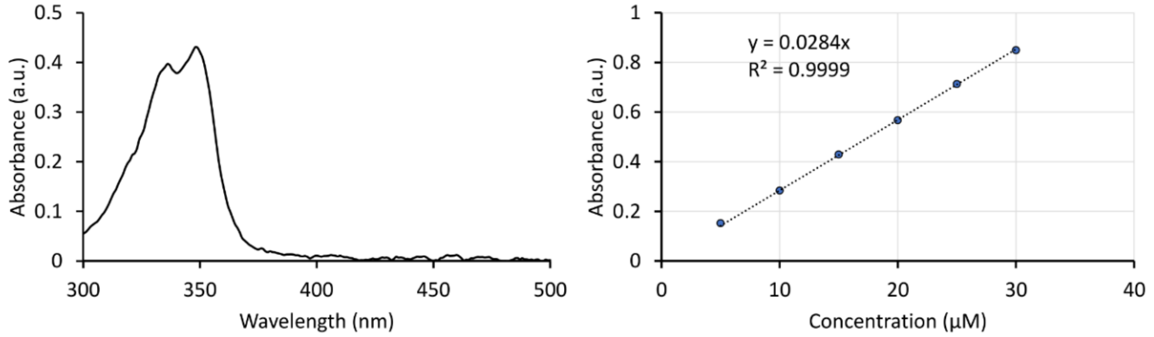

Figure S12. *UV-VIS spectrum of a 15 µM solution of QZ* ***C4*** *in toluene and determination of the extinction coefficient of QZ* ***C4*** *at 348 nm (ε=28 400 dm^{3} mol^{-1} cm^{-1}).*

Spectrum and extinction coefficient of QZ C8

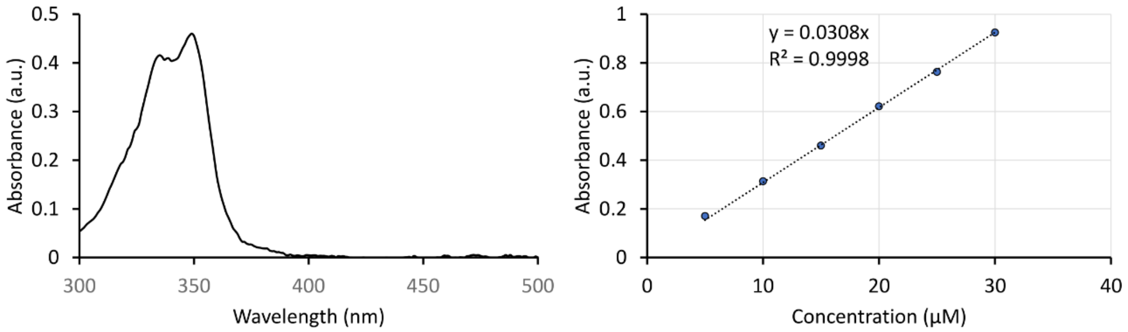

Figure S13*. UV-VIS spectrum of a 15 µM solution of QZ* ***C8*** *in toluene and determination of the extinction coefficient of QZ* ***C8*** *at 349 nm (ε=30 800 dm^{3} mol^{-1} cm^{-1}).*

Spectrum and extinction coefficient of QZ C12

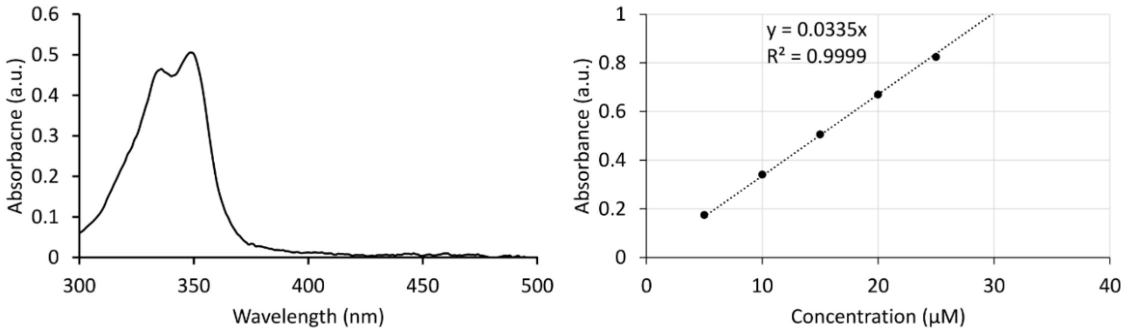

Figure S14*. UV-VIS spectrum of a 15 µM solution of QZ* ***C12*** *in toluene and determination of the extinction coefficient of QZ* ***C12*** *at 349 nm (ε=33 500 dm^{3} mol^{-1} cm^{-1}).*

Spectrum and extinction coefficient of QZ C16

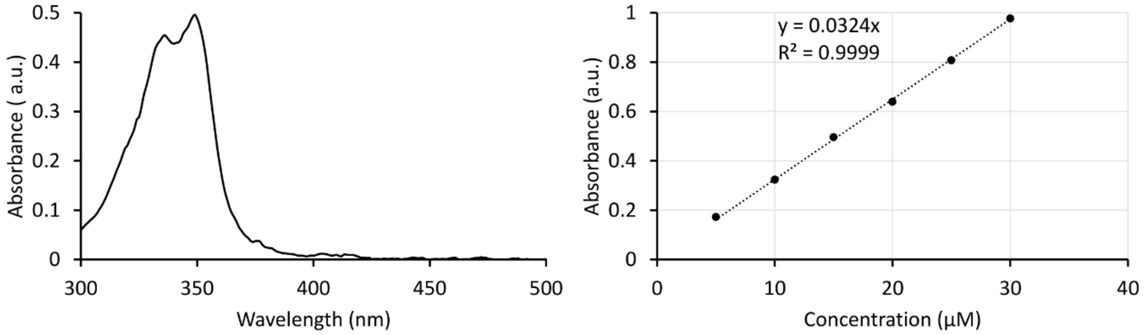

Figure S15. *UV-VIS spectrum of a 15 µM solution of QZ **C16** in toluene and determination of the extinction coefficient of QZ **C16** at 349 nm (ε=32 400 dm^3 mol^{-1} cm^{-1}).*

Spectrum and extinction coefficient of QZ NP

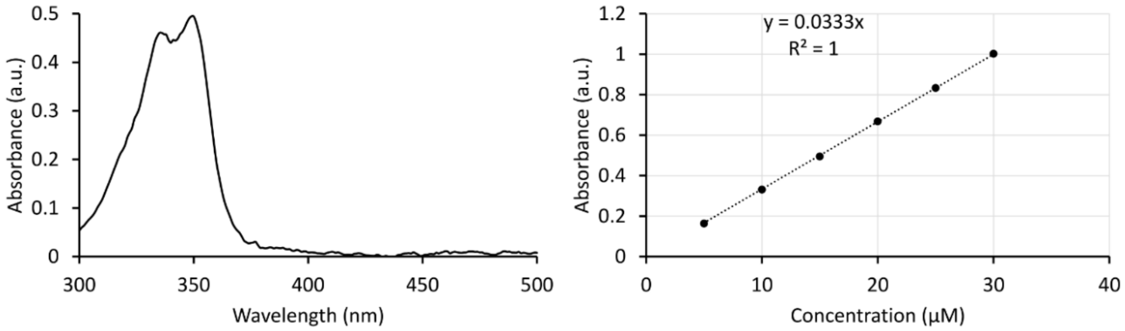

Figure S16. *UV-VIS spectrum of a 15 µM solution of QZ **NP** in toluene and determination of the extinction coefficient of QZ **NP** at 349 nm (ε=33 300 dm^3 mol^{-1} cm^{-1}).*

Spectrum and extinction coefficient of QZ C4 + acid

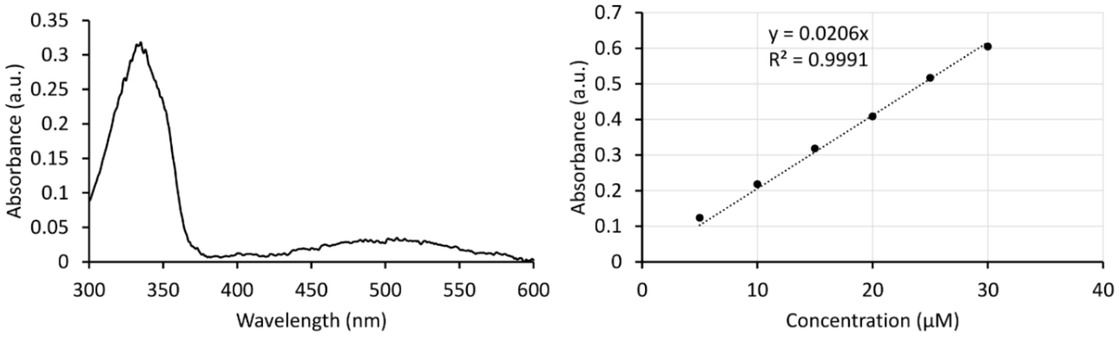

Figure S17. *UV-VIS spectrum of a 15 µM solution of QZ **C4** in DCM with excess benzenesulfonic acid (12 equivalents) and determination of the extinction coefficient of QZ **C4** at 335 nm (ε=20 600 dm^3 mol^{-1} cm^{-1}).*

Spectrum and extinction coefficient of QZ C8 + acid

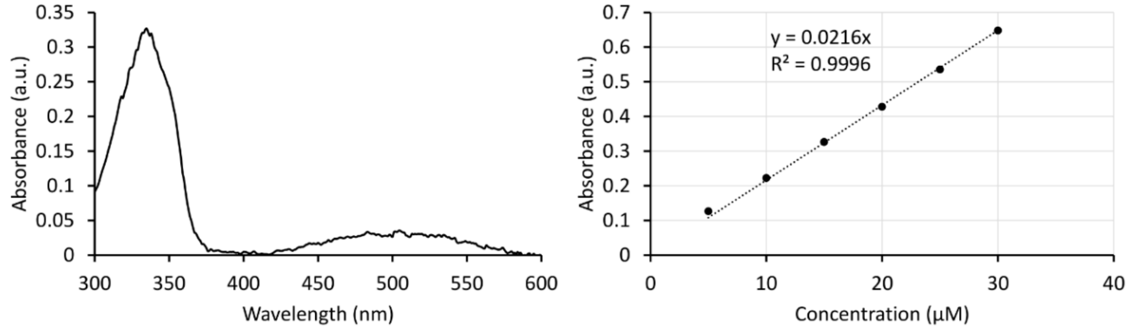

***Figure S18**. UV-VIS spectrum of a 15 μM solution of QZ **C8** in DCM with excess benzenesulfonic acid (12 equivalents) and determination of the extinction coefficient of QZ **C8** at 335 nm (ε=21 600 dm^3 mol^{-1} cm^{-1}).*

Spectrum and extinction coefficient of QZ C12 + acid

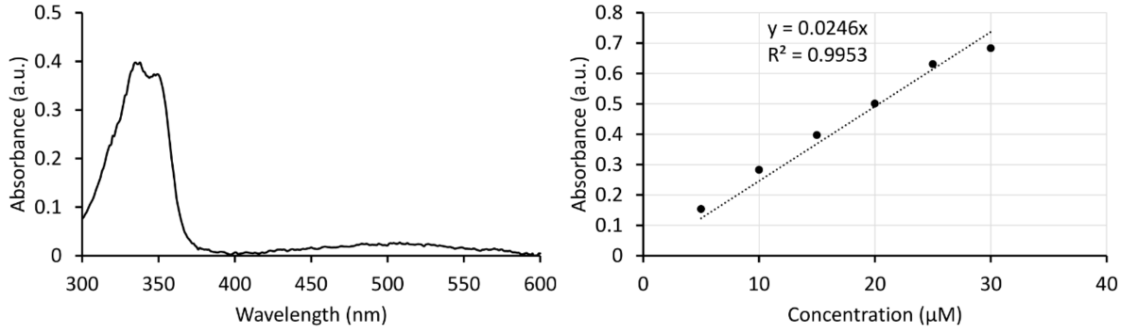

***Figure S19.** UV-VIS spectrum of a 15 μM solution of QZ **C12** in DCM with excess benzenesulfonic acid (12 equivalents) and determination of the extinction coefficient of QZ **C12** at 335 nm (ε=24 600 dm^3 mol^{-1} cm^{-1}).*

Spectrum and extinction coefficient of QZ C16 + acid

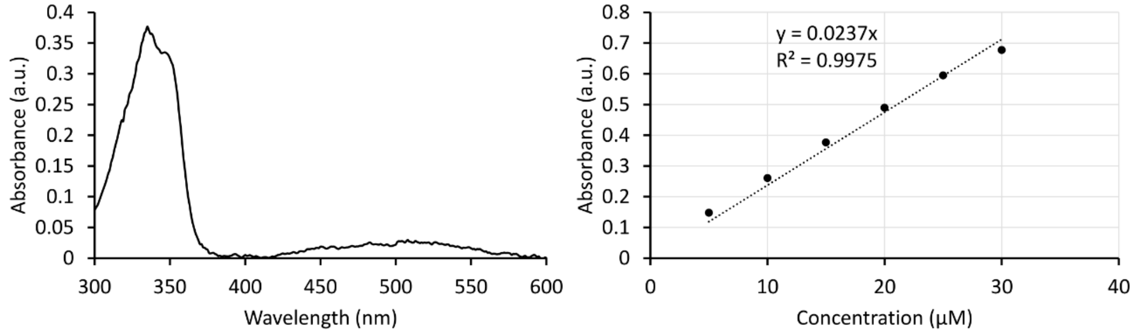

***Figure S20.** UV-VIS spectrum of a 15 μM solution of QZ **C16** in DCM with excess benzenesulfonic acid (12 equivalents) and determination of the extinction coefficient of QZ **C16** at 335 nm (ε=23 700 dm^3 mol^{-1} cm^{-1}).*

Spectrum and extinction coefficient of QZ NP + acid

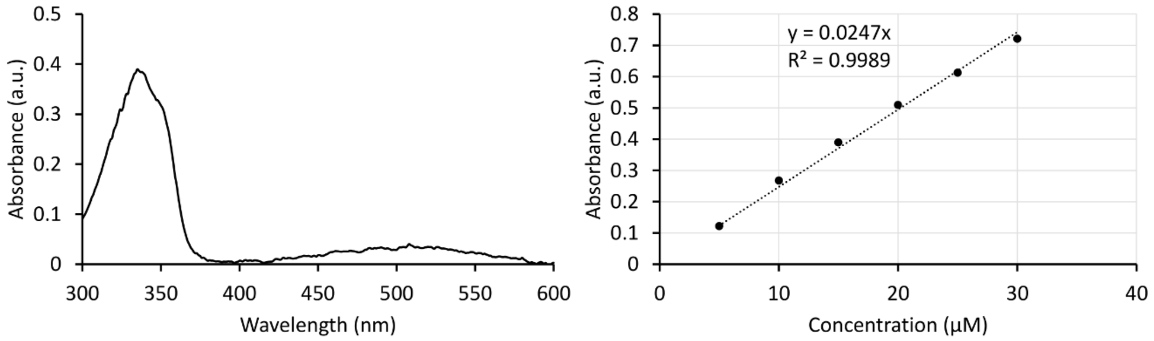

***Figure S21**. UV-VIS spectrum of a 15 µM solution of QZ **NP** in DCM with excess benzenesulfonic acid (12 equivalents) and determination of the extinction coefficient of QZ **NP** at 335 nm (ε=24 700 dm^3 mol^{-1} cm^{-1}).*

Conclusion extinction coefficients

Differences in extinction coefficient were observed while this was not expected for QZ with different chain lengths as their conjugated part is not significantly influenced by the difference in chain length. To determine the amount of adsorption and separation below, 33 400 dm^3 mol^{-1} cm^{-1} is used as extinction coefficient for the amount of adsorption and 24 600 dm^3 mol^{-1} cm^{-1} for the amount of separation. The validity of this assumption will be discussed below.

5. Bulk adsorption and washing experiments

General experimental procedure

To test the adsorption in bulk, different molecules and different graphitic powders were tested. Three experiments were performed together to minimize errors. ^{1}H-NMR spectra were acquired in $CDCl_3$ on a Bruker AMX 400 MHz and chemical shifts (δ) are reported in parts per million (ppm) referenced to tetramethylsilane. UV/VIS absorption spectra were recorded on a PerkinElmer Lambda950 spectrophotometer using blank correction. Dye solutions were prepared using commercially available dichloromethane and toluene of at least spectro or HPLC grade.

Adsorption of pure compounds on graphitic powders

The reaction tubes were flame dried and cooled in a dessicator. The graphitic powders were dried in a vacuum oven at 50 °C overnight. The QZ was dissolved in 14 mL dry toluene with a concentration of 3 mM and the solution was sonicated under nitrogen atmosphere. 10 mg of the different graphitic powders were added to 10 mL reaction tubes and one empty reaction tube was used as blank. The reaction tubes were evacuated on a Schlenk line and back-filled with nitrogen. To each reaction tube, 2 mL of the solution was added. The mixtures were stirred for 24 hours at 60 °C. After this time, the graphite was removed *via* centrifugation and washed with toluene. The solution was diluted to 5 mL, then diluted 80x more and measured with UV/VIS spectroscopy.

Adsorption of mixtures on GO, rGO, GNP and deactivated GNP

The reaction tubes were flame dried and cooled in a dessicator. The graphitic powders were dried in the vacuum oven at 50 °C overnight. QZ **C12** and QZ **NP** were dissolved in 9 mL dry toluene both with a concentration of 1.5 mM and the solution was sonicated under nitrogen atmosphere. 10 mg graphitic powder was added to three empty 10 mL reaction tubes and one empty reaction tube was used as blank. The reaction tubes were evacuated on a Schlenk line and back-filled with nitrogen. To each reaction tube, 2 mL of the solution was added. The mixtures were stirred for 24 hours at 60 °C. After this time, the graphitic powder was removed *via* centrifugation and washed with toluene. The solution was diluted to 5 mL, then diluted 80x more and characterized with UV/VIS spectroscopy. The remaining solution was evaporated under reduced pressure and measured with ^{1}H-NMR spectroscopy. The powder was washed 3 times with 2 mL of a saturated solution of benzene sulfonic acid in DCM. The washed solution were collected and filtered through a paper filter to remove rests of powder and diluted to 20 mL. The solution was diluted 10x and characterized with UV/VIS spectroscopy. The remaining solution was extracted with sat. $NaHCO_3$ and sat. NaCl, dried with Na_2SO_4 and evaporated under reduced pressure. The residue was measured with ^{1}H-NMR spectroscopy.

Adsorption of mixtures on graphite (example for QZ C12 and QZ NP)

Because of the low surface area of graphite, larger amounts of graphite were necessary to see a significant effect on the solution composition. When higher amounts of another powder were used, the amount of QZ left in solution was negligible and no selecitivity in adsorption was observed.

The reaction tubes were flame dried and cooled in a dessicator. Graphite was dried in the vacuum oven at 50 °C overnight. QZ **C12** and QZ **NP** were dissolved in 90 mL dry toluene both with a concentration of 0.15 mM and the solution was sonicated under nitrogen atmosphere. 500 mg graphite was added to three empty 100 mL reaction tubes and one empty reaction tube was used as blank. The reaction tubes were evacuated on a Schlenk line and back-filled with nitrogen. To each reaction tube, 20 mL of the solution was added. The mixtures were stirred for 24 hours at 60 °C. After this time, the graphite was removed *via* centrifugation and washed with toluene. The solution was diluted to 100 mL, then diluted 4x more and measured with UV/VIS spectroscopy. The remaining solution was evaporated under reduced pressure and measured with ^{1}H-NMR spectroscopy. The powder was washed 3 times with 5 mL of a saturated solution of benzene sulfonic acid in DCM. The separated solution was collected and filtered through a paper filter to remove rests of graphite and diluted to 20 mL. The solution was diluted 5x and characterized with UV/VIS spectroscopy. The remaining solution was extracted with sat. $NaHCO_3$ and sat. NaCl, dried with Na_2SO_4 and evaporated under reduced pressure. The residue was measured with ^{1}H-NMR spectroscopy.

Determination of the adsorption selectivity

The absolute amounts of both QZ **C12** and QZ **NP** in the different solutions were determined by using the peak at 349 nm in UV/VIS and the general extinction coefficient and the relative ratio of the peaks at 5.49 (QZ **C12**) and 5.51 (QZ **NP**) ppm in the ^{1}H-NMR spectrum. These amounts were compared to the amounts present in the blank. From these values, the amount of adsorbed molecules can be calculated by subtracting the amount in solution from the total amount given by the blank. The adsorption selectivity is then defined as the amount of adsorbed QZ **C12** divided by the total amount of adsorbed QZ (**C12+NP**).

For the example shown below (Figure S22 and S23):

An absorbance of 0.22 was determined in comparison to an absorbance of 0.54 for the blank. Using the general extinction coefficient and the relative amounts in the ^{1}H-NMR spectrum, it can be calculated that the blank contains 3.44 µmol of QZ **C12** and 3.05 µmol of QZ **NP**. The solution after adsorption contains 0.98 µmol of QZ **C12** and 1.60 µmol of QZ **NP**. This means that respectively

2.46 µmol of QZ **C12** and 1.45 µmol of QZ **NP** has been adsorbed. This gives an adsorption selectivity of 60%.

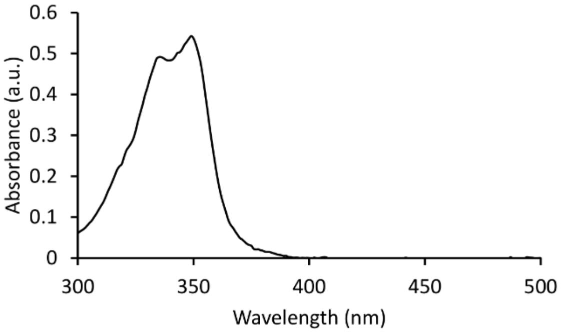

Figure S22. *UV-VIS spectrum of the blank (left) and the remaining solution after centrifugation of the graphitic powder (right). (Example of deactivated GNP in combination with QZ **C12** and QZ **NP**)*

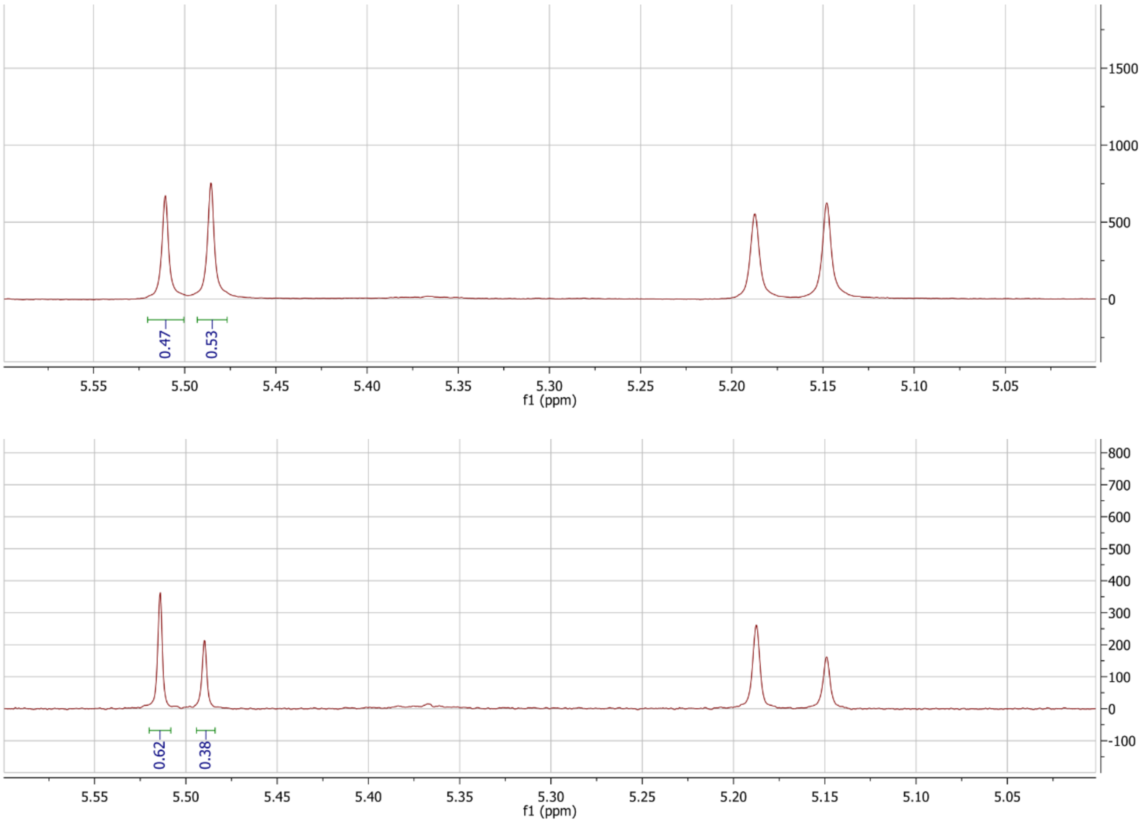

Figure S23*. ^{1}H-NMR spectrum of the blank (up) and of the remaining solution after centrifugation of the graphitic powder (bottom). (Example of deactivated GNP in combination with QZ **C12** and QZ **NP**)*

Determination of the separation selectivity

The absolute amounts of both QZ **C12** and QZ **NP** in the different solutions were determined by using the peak at 335 nm in UV/VIS and the general extinction coefficient and the relative ratio of

the peaks at 5.49 (QZ **C12**) and 5.51 (QZ **NP**) ppm in ^{1}H-NMR. These amounts were corrected for the amounts present in the blank. The separation selectivity is defined as the amount of desorbed QZ **C12** divided by the total amount of desorbed QZ (**C12+NP**).

For the example shown below (Figure S24 and S25):

An absorbance of 0.14 was determined for the separated solution. Using the general extinction coefficient and the relative amounts in the ^{1}H-NMR spectrum, it can be calculated that the separated solution contains 0.82 µmol of QZ **C12** and 0.19 µmol of QZ **NP**. This gives a separation selectivity of 81%.

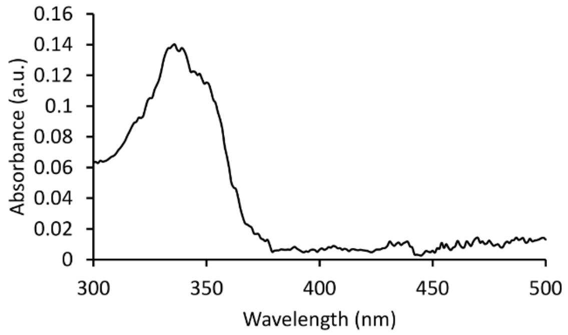

Figure S24. *UV-VIS spectrum of the separated solution. (Example of deactivated GNP in combination with QZ **C12** and QZ **NP**)*

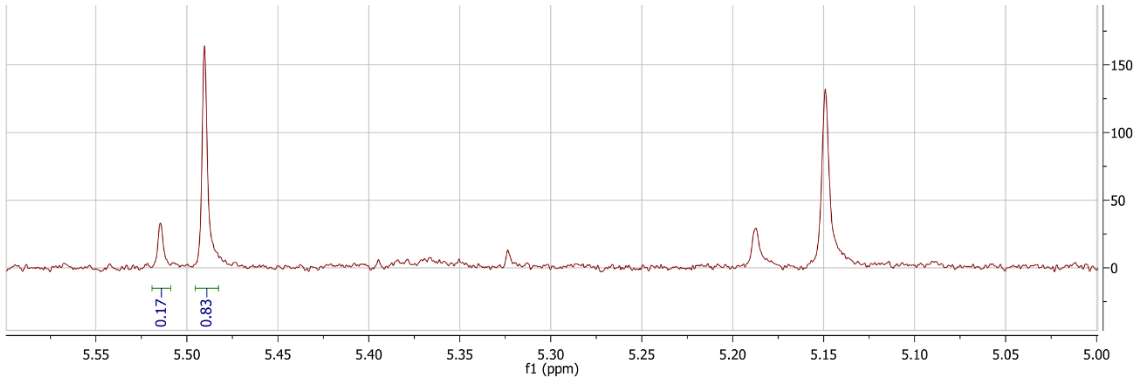

Figure S25. *^{1}H-NMR spectrum of the separated solution. (Example of deactivated GNP in combination with QZ **C12** and QZ **NP**)*

Recycling experiments

In the recycling experiments, the same protocol is followed as described in 'Adsorption of mixtures on graphite'. After washing the graphitic powder with saturated benzene sulfonic acid in DCM. The graphitic powder is washed three times with 10 mL of a 95/5 solution of methanol/triethylamine and dried overnight at 50°C in a vacuum oven. This powder is then reused as graphite powder following the same protocol.

Summary of the obtained results

Below a summary is given of the results that were obtained for different QZs and different graphitic powders. To validate our assumption that a general extinction coefficient could be used, both results with a general extinction coefficients and the specific extinction coefficients for every QZ are given. Since there are only minor differences between both tables (indicated in yellow), we can conclude that this assumption is valid.

Table S3. *Summary of the adsorption amounts and adsorption selectivity, and washing amounts and washing selectivity under different conditions. Values are calculated using general extinction coefficients of 33 400 dm^3 mol^{-1} cm^{-1} to determine the adsorbed amounts and of 24 600 dm^3 mol^{-1} cm^{-1} to determine the separated amounts. No values are indicated for the adsorption of cycle 6 and 7 as the characteristic ^{1}H-NMR peak was not present in the solution of non-adsorbed molecules. However, the characteristic peak in UV/VIS was present.*

QZ's	powder	Amount adsorbed QZ alkyl chain(%)	Amount adsorbed QZ NP (%)	Adsorption selectivity (%)
C12+NP	GO	53±11	51±11	50.8±0.2
C12+NP	rGO	86±4	58±15	60±5
C12+NP	GNP	73±2	48±5	60±2
C12+NP	Deactivated GNP	60±9	32±14	66±7
C12+NP (cycle 1)	Graphite (500 mg)	79±2	6±1	93±1
C12+NP (cycle 2)	Graphite (500 mg)	66±2	18±13	80±12
C12+NP (cycle 3)	Graphite (500 mg)	70±2	11±5	86±5
C12+NP (cycle 4)	Graphite (500 mg)	80±3	19±3	81±2
C12+NP (cycle 5)	Graphite (500 mg)	100±0	100±0	50±0
C12+NP (cycle 6)	Graphite (500 mg)	/	/	/
C12+NP (cycle 7)	Graphite (500 mg)	/	/	/
C4+NP	Graphite (500 mg)	26±2	14±4	66±8
C8+NP	Graphite (500 mg)	54±8	28±10	66±4
C16+NP	Graphite (500 mg)	96±1	14±3	88±2

QZ's	powder	Amount separated QZ alkyl chain (%)	Amount separated QZ NP (%)	Separation selectivity (%)
C12+NP	GO	3.8±0.3	16±1	19±2
C12+NP	rGO	45±10	21±1	68±4
C12+NP	GNP	31±7	11±2	73±2
C12+NP	Deactivated GNP	40±14	9±3	82±2
C12+NP (cycle 1)	Graphite (500 mg)	41±1	1.1±0.3	97.5±0.6
C12+NP (cycle 2)	Graphite (500 mg)	32±1	1±1	98±2
C12+NP (cycle 3)	Graphite (500 mg)	24±2	0.3±0.3	99±1
C12+NP (cycle 4)	Graphite (500 mg)	27±1	0.7±0.1	97±0
C12+NP (cycle 5)	Graphite (500 mg)	75±11	24±10	75±10
C12+NP (cycle 6)	Graphite (500 mg)	36±4	0.7±0.3	98±1

C12+NP (cycle 7)	Graphite (500 mg)	30±1	1±1	98±2
C4+NP	Graphite (500 mg)	/	/	/
C8+NP	Graphite (500 mg)	7.0±0.3	0.3±0.4	96±5
C16+NP	Graphite (500 mg)	45±9	0.7±0.3	98.6±0.5

Table S4. *Summary of the adsorption amounts and selectivity and washing amounts and selectivity under different conditions. Values are calculated using the characteristic extinction coefficient of each QZ. No values are indicated for the adsorption of cycle 6 and 7 as the characteristic ^{1}H-NMR peak was not present in the solution of non-adsorbed molecules. However, the characteristic peak in UV/VIS was present.*

QZ's	powder	Amount adsorbed QZ alkyl chain(%)	Amount adsorbed QZ NP (%)	Adsorption selectivity (%)
C12+NP	GO	53±11	51±10	50.8±0.2
C12+NP	rGO	86±4	58±14	60±5
C12+NP	GNP	74±2	49±5	60±2
C12+NP	Deactivated GNP	60±9	32±14	66±7
C12+NP (cycle 1)	Graphite (500 mg)	79±2	6±1	93±1
C12+NP (cycle 2)	Graphite (500 mg)	66±2	18±13	80±12
C12+NP (cycle 3)	Graphite (500 mg)	70±2	12±5	86±5
C12+NP (cycle 4)	Graphite (500 mg)	80±3	19±3	81±2
C12+NP (cycle 5)	Graphite (500 mg)	100±0	100±0	50±0
C12+NP (cycle 6)	Graphite (500 mg)	/	/	/
C12+NP (cycle 7)	Graphite (500 mg)	/	/	/
C4+NP	Graphite (500 mg)	26±2	14±4	65±8
C8+NP	Graphite (500 mg)	54±8	29±10	66±4
C16+NP	Graphite (500 mg)	97±1	15±3	87±2

QZ's	powder	Amount separated QZ alkyl chain (%)	Amount separated QZ NP (%)	Separation selectivity (%)
C12+NP	GO	3.8±0.3	16±1	19±2
C12+NP	rGO	45±9	21±1	68±4
C12+NP	GNP	31±7	11±1	73±2
C12+NP	Deactivated GNP	40±14	9±3	82±2
C12+NP (cycle 1)	Graphite (500 mg)	41±1	1.1±0.3	97.5±0.6
C12+NP (cycle 2)	Graphite (500 mg)	32±1	1±1	98±2
C12+NP (cycle 3)	Graphite (500 mg)	24±1	0.3±0.3	99±1
C12+NP (cycle 4)	Graphite (500 mg)	27±1	0.7±0.1	97±0
C12+NP (cycle 5)	Graphite (500 mg)	75±13	24±10	75±10
C12+NP (cycle 6)	Graphite (500 mg)	36±2	0.7±0.3	98±1
C12+NP (cycle 7)	Graphite (500 mg)	30±1	1±1	98±2
C4+NP	Graphite (500 mg)	/	/	/
C8+NP	Graphite (500 mg)	7.6±0.4	0.3±0.4	96±5
C16+NP	Graphite (500 mg)	46±9	0.7±0.3	98.6±0.5

Sources:

1. Q. Z. Yang, O. Siri and P. Braunstein, *Chem. Commun.*, 2005, **5**, 2660–2662.
2. A. Luczak, A. T. Ruiz, S. Pascal, A. Adamski, J. Jung, B. Luszczynska and O. Siri, *Polymers*, 2021, **13**, 1567.
3. Y. Fang, N. Salamé, S. Woo, D. S. Bohle, T. Friščić and L. A. Cuccia, *CrystEngComm*, 2014, **16**, 7180–7185.
4. Y. Fang, M. Cibian, G. S. Hanan, D. F. Perepichka, S. De Feyter, L. A. Cuccia and O. Ivasenko, *Nanoscale*, 2018, **10**, 14993–15002.
5. O. Siri and P. Braunstein, *Chem. Commun.*, 2002, **2**, 208–209.
6. H. Ren, E. Cunha, Q. Sun, Z. Li, I. A. Kinloch, R. J. Young and Z. Fan, *Nanoscale Adv.*, 2019, **1**, 1432–1441.
7. N. Fairley, V. Fernandez, M. Richard-Plouet, C. Guillot-Deudon, J. Walton, E. Smith, D. Flahaut, M. Greiner, M. Biesinger, S. Tougaard, D. Morgan and J. Baltrusaitis, *Applied Surface Science Advances*, 2021, **5**, 100112.
8. J. Walton, M. R. Alexander, N. Fairley, P. Roach and A. G. Shard, *Surf Interface Anal*, 2016, **48**, 164-172.
9. A. G. Shard, *J. Vac. Sci. Technol. A*, 2020, **38**, 041201.
10. M.P. Seah, *Surf. Interface Anal.*, 2012, **44**, 1353-1359.
11. M.P. Seah, *J. Electron Spectrosc. Relat. Phenom.*, 1995, **71**, 191-204,
